# 3D-printed nHA/PA66 porous scaffold: Regulating immune balance and vascularization synergistically promotes bone regeneration^☆^^☆☆^

**DOI:** 10.1016/j.mtbio.2025.102315

**Published:** 2025-09-14

**Authors:** Caiping Yan, Fukang Zhu, Hao Liang, Changxing Liu, Bin He, Taiyou Wang, Heling Tan, Hong Li, Dianming Jiang, Bo Qiao

**Affiliations:** a Department of Orthopedics, The First Affiliated Hospital of Chongqing Medical University, 1 Youyi Rd, Chongqing, 400010, China; b Department of Orthopedics, Laboratory of Biological Tissue Engineering and Digital Medicine, Affiliated Hospital of North Sichuan Medical College, No. 1 The South of Maoyuan Road, Nanchong, Sichuan, 637000, China; c Chongqing Municipal Health Commission Key Laboratory of Musculoskeletal Regeneration and Translational Medicine, the first affiliated hospital of Chongqing Medical University, China; d Department of Orthopedics, The Third Affiliated Hospital of Chongqing Medical University, Chongqing, China; e College of Physics, Sichuan University, Chengdu, 610065, China

**Keywords:** Critical-sized bone defect, Nano-hydroxyapatite/polyamide 66 (nHA/PA66), 3D printing, Arburg plastic free-forming (APF), Immunoregulation, Bone regeneration

## Abstract

3D-printed porous scaffolds have emerged as a potential solution for treating critical-sized bone defects, yet localized inflammatory imbalance leads to suboptimal therapeutic outcomes. This study utilized nHA/PA66 composite material as the foundation, employing APF additive manufacturing technology to fabricate a three-dimensional porous scaffold (HP) suitable for local bone filling, and functionalized the porous scaffold through modification with polydopamine and magnesium ions (Mg^2+^) (HPDM). First, the physicochemical properties and biosafety of the HPDM scaffold were verified, followed by a comparative analysis of its effects on macrophage phenotype transformation, vascular endothelial cell differentiation, and pre-osteoblast differentiation differences. The HPDM scaffold achieves coordinated balance in the transformation between M1 and M2 macrophages through sustained release of polydopamine and Mg^2+^. Mg^2+^ plays a crucial role in inflammatory regulation by downregulating the NF-κB signaling pathway. Furthermore, several experiments demonstrated that the HPDM scaffold regulates orderly inflammatory responses to promote intercellular interactions, stimulating angiogenesis and osteogenic regeneration. In New Zealand rabbits' femoral condyle bone defect model, the HPDM porous scaffold achieved significant vascularized bone regeneration. This study confirms that the functionalized HPDM porous scaffold prepared using nHA/PA66 as the base material has significant potential in regulating immune responses and enhancing vascularized bone regeneration.

## Highlights

• Porous nHA/PA66 scaffolds with micron-sized pores were fabricated using the “melt deposition method' and APF printing technology, demonstrating certain advantages in bioactivity, mechanical strength, and promoting bone regeneration in vivo.
• The —HPDM— scaffold exhibits the following characteristics: convenient fabrication, excellent cell adhesion, superior biosafety, regulation of macrophage polarization, ROS scavenging, immune microenvironment remodeling, —promotion of vascular and bone regeneration—.
• The HPDM scaffold induces macrophage polarization towards the M2 phenotype by inhibiting the NF-κB signaling pathway while simultaneously activating the TGF-β1 and BMP-2 signaling pathways to promote osteogenesis.
• The HPDM scaffold facilitates endothelial cell differentiation, engages in crosstalk with osteoblasts via the Notch signaling pathway, suppresses osteoclast activity, regulates the balance of bone metabolism, and accomplishes regenerative repair of critical bone defects.

## Graphical abstract

**Figure:**
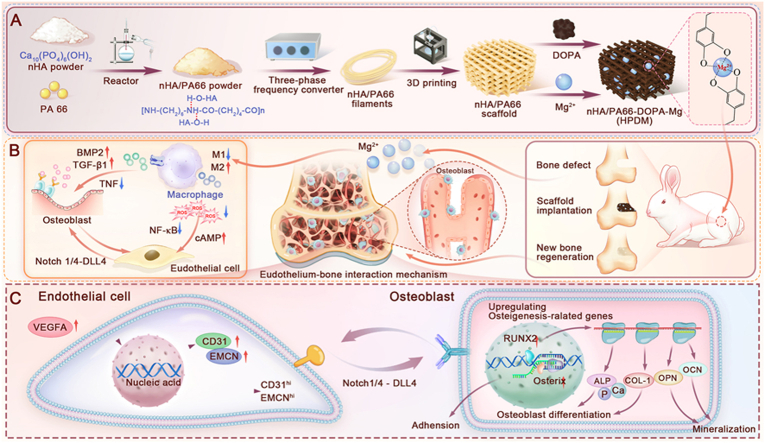

## 1 Introduction

Critical-sized bone defect (CSD) refers to the smallest size of bone defect that cannot heal naturally, commonly observed in cases such as traumatic bone loss, post-tumor resection, and infected nonunion [1,2]. Current treatment approaches primarily include bone grafting (using autologous or allogeneic bone), bone transport (via the Ilizarov technique), biomaterial filling (such as hydroxyapatite and collagen scaffolds), and growth factor supplementation [3,4]. However, the therapeutic outcomes remain unsatisfactory, making this disease one of the leading causes of high disability and mortality rates.

In recent years, the rapid development of biological tissue engineering materials has brought new hope for treating CSD. The nHA/PA66 composite material is a commonly used clinical artificial bone graft substitute [5]. Not only does it possess a composition and structure similar to the bone matrix, but it also exhibits excellent biocompatibility, mechanical properties, osteoinductivity, and osteoconductivity. However, the traditional processing techniques for nHA/PA66 composites mainly involve injection molding and CO_2_ foaming, resulting in a dense product structure that significantly differs from the microscopic morphology of normal human bone tissue. This is unfavorable for bone ingrowth and substitution, limiting its clinical applications. 3D printing technology enables precise control over the material's structure, pore size, and mechanical properties, further enhancing its clinical applicability. However, the characteristics of nHA/PA66, such as high strength, high melting point, and the tendency of nHA to agglomerate, impose higher requirements on 3D printing. The freeform 300-3X 3D printer produced by ARBURG features unique Arburg Plastic Freeforming (APF) technology, which enables personalized processing of high-strength plastic-based granules through droplet size adjustment and parameter control. Currently, no literature reports using APF technology for processing nHA/PA66, whereas this study successfully achieved precise 3D printing of nHA/PA66 using APF technology.

In most cases, implanting bone tissue engineering materials into the body triggers localized inflammatory responses. The acute inflammatory phase is primarily characterized by neutrophil infiltration, which subsequently evolves into chronic inflammation dominated by macrophage infiltration [6,7], and induces fibrotic hyperplasia around and inside the implant, hindering bone formation. Macrophages exhibit three phenotypes: M0, M1, and M2. M1 macrophages primarily function in clearing necrotic tissues and activating host defense mechanisms, while M2 macrophages mainly promote tissue repair [8]. During the bone tissue repair, the M1/M2 polarization imbalance within the tissue increases M1 macrophages and their fusion to form multinucleated foreign body giant cells (FBGCs). This subsequently promotes the growth of fibrous scar tissue around the biomaterial or the infiltration of fibroblasts into the scaffold, thereby hindering bone tissue regeneration within the scaffold [9].

Moreover, vascular formation and bone formation are mutually coordinated during the repair process of bone defects. The ingrowth of new blood vessels is a prerequisite for bone tissue formation. Studies have reported that since oxygen and nutrients in bone tissue cannot act on osteoblasts through long-distance diffusion, and only osteoblasts adjacent to vascular tissues can survive effectively, the growth rate and extent of neovascularization are key determinants of new bone formation [[10], [11], [12]]. After implantation of bone repair materials into bone defect sites, the immune response generated by local macrophages includes both foreign body reactions to the material and participation in angiogenesis and vascular remodeling within the bone repair material. By regulating the activation of macrophages with different phenotypes and the secretion of pro-angiogenic factors, promoting vascular regeneration and bone repair represents a viable tissue engineering strategy [13,14]. Therefore, regulating macrophage polarization through surface modification of biomaterials is currently one of the new directions in the design of bone tissue biomaterials.

Mg^2+^ is the fourth most abundant cation in the human body and plays important roles in macrophage polarization regulation [15], angiogenesis promotion [16,17], and osteogenesis acceleration [18,19]. Immobilizing Mg^2+^ on the surface of scaffold materials is an effective strategy to enhance the bioactivity of scaffold materials [20,21]. Polydopamine (PDA) coating exhibits excellent biocompatibility, and its abundant functional groups, such as amino and imino groups, can serve as “bridges' to react with other compounds [22]. Therefore, PDA can form a complete and dense activated layer on the surface of the nHA/PA66 porous scaffold, providing a reactive foundation for further modification with Mg^2+^. Beyond its universal adhesive properties and abundant amino groups, PDA possesses unique advantages including antioxidant activity, fluorescence quenching capability, and excellent biocompatibility—particularly in terms of antioxidant activity. Consequently, utilizing PDA coatings provides a flexible modification platform for constructing multifunctional scaffold materials while simultaneously leveraging its antioxidant properties, achieving the dual benefit of killing two birds with one stone [23,24].

**Scheme 1:**
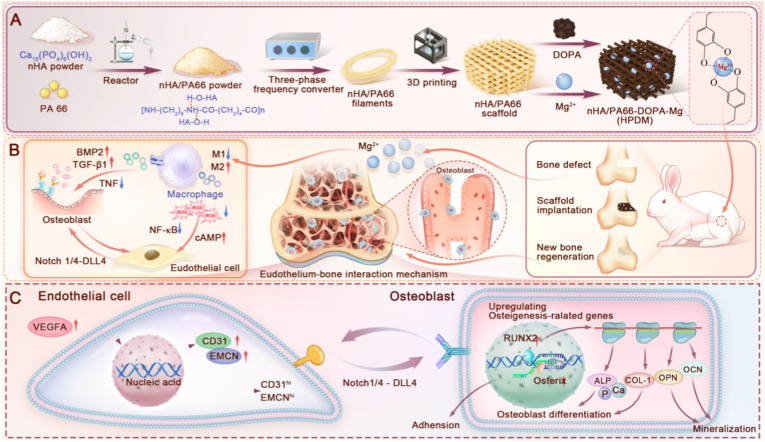
Schematic diagram of HPDM porous scaffold preparation and its regulation of osteoimmunology to promote vascularized bone regeneration. (A) The preparation process of nHA/PA66 raw materials, porous scaffolds, and their functional modifications (loading Mg^2+^). (B) Application of HPDM Porous Scaffold in New Zealand Rabbit Bone Defect Model and Its Mechanism of Regulating Ordered Inflammatory Response to Promote Cellular Interactions, Stimulating Angiogenesis and Osteogenic Regeneration. (C) The mechanism by which endothelial cells of the newly formed “H"-type blood vessels in HPDM porous scaffolds interact with surrounding osteoblasts to promote bone regeneration.

In this study, we processed nHA/PA66 composite materials using APF technology to prepare nHA/PA66 porous scaffolds with a microporous structure. On this basis, the nHA/PA66 porous scaffold was functionalized with polydopamine and Mg^2+^ (HPDM) to enhance its ability to regulate local macrophage polarization and promote vascular regeneration. Through material characterization, macrophage polarization effect identification, and in vitro and in vivo pro-angiogenic osteogenic function validation, it was successfully demonstrated that the nHA/PA66 scaffold modified with Mg^2+^ and polydopamine can effectively regulate the immune response in bone defect areas, inhibit excessive fibrotic hyperplasia, and promote vascularized bone regeneration (Scheme 1).

## 2 Methods

### 2.1 Preparation of Mg^2+^-modified nHA/PA66 porous scaffold (HPDM)

Weigh PA66 and CaCl_2_ in a mass ratio 1:2 (w/w), add them into the reaction vessel, introduce anhydrous ethanol, and heat with stirring (85–95 °C, 1000 r/min) until completely dissolved. Subsequently, nHA slurry was added, and stirring was continued for 2 h (85–95 °C, 1500 r/min) to ensure thorough compounding of nHA with PA66 before terminating the reaction. The mixture was then cooled, washed, and dried to obtain the nHA/PA66 composite material. The nHA/PA66 composite material was pulverized using a Planetary ball mill (China, Shanghai) at 3500 r/min, followed by wire drawing with a Three-phase variable-frequency twin-screw extruder (China, Shanghai) at temperatures of 240 °C, 265 °C, and 285 °C to prepare approximately 5 mm long “Cylindrical-pellets.' Place the nHA/PA66 “Cylindrical-pellets' into the hopper of the 3D print machine (ARBURG, freeformer 300-3X), and prepare porous scaffolds with a pore size of 400 μm using APF technology (Scheme 1A) [25,26]. Then, immerse the nHA/PA66 porous scaffold into a mixed solution containing 10 mM Tris and 2 mM dopamine (pH 8.5, 25 °C) and allow the reaction to proceed continuously for 24 h in the dark until the scaffold surface is completely covered with polydopamine. The porous scaffold was rinsed with ultrapure water 3 times (5 min each time) and thoroughly dried. It was then immersed in a reaction solution containing 10 mM Tris and 50 mg/mL MgCl_2_ (pH 8.5, 25 °C) for 24 h (60 r/min). Rinse the porous scaffold with distilled water 3 times (5 min each time), then dry it for standby use (Fig. S1).

The unmodified nHA/PA66 porous scaffold was designated as the “HP' group, the nHA/PA66 porous scaffold modified solely with polydopamine was designated as the “HPD' group, the nHA/PA66 porous scaffold modified with both polydopamine and Mg^2+^ was designated as the “HPDM' group (Fig. S2).

### 2.2 Characterization and analysis of porous scaffolds

To compare the differences among the three scaffold materials in terms of microstructure, surface roughness, hydrophilicity, and chemical bonding modes, we employed various characterization techniques, including Scanning Electron Microscope (SEM) (Germany, Zeiss), Atomic Force Microscope (AFM) (Germany, Bruker), X-ray Diffraction (XRD) (England, Malvern Panalytical), Fourier Transform Infrared Spectroscopy (FTIR) (Germany, Bruker), and Video-based Contact Angle Goniometer (Germany, DataPhysics). All tests were repeated three times.

To investigate the effect of modification, the compressive mechanical properties of nHA/PA6 porous scaffolds before and after modification were evaluated in accordance with international standards GB/T 1041-92. Each sample was tested in triplicate.

### 2.3 Porous scaffold degradation experiment

To mimic the in vivo microenvironment, we constructed a dynamic degradation device (Fig. S3) for the degradation experiment of porous scaffolds [27]. The porous scaffold was fixed in this device and continuously flushed with circulating degradation solution (Hanks solution containing 100U/mL lipase, pH 7.4) at a 1 mL/min flow rate for 8 weeks. The degradation solution was collected using an Erlenmeyer flask. After the degradation of the porous scaffold, the supernatant solution was collected, and the Mg^2+^ concentration was measured using an atomic absorption spectrophotometer (Japan, Shimadzu). The Mg^2+^ loading rate of the HPDM scaffold was then calculated according to formula (1):

(1) Loadrate=[(Initialconcentration‐Residualconcentration)/Initialconcentration]×100%

Prepare the HPDM scaffold extract according to the national standard GB/T16886.12–2017, measure the Mg^2+^ concentration in the extract, and plot the Mg^2+^ release curve. Immerse the scaffold in D-Hanks solution (without Mg^2+^) at a ratio of 1 g:10 mL for 15 days, replacing the D-Hanks solution every 2 days, and measure the Mg^2+^ concentration using atomic absorption spectrophotometry. Then, plot the cumulative release curve of Mg^2+^ and analyze the Mg^2+^ release characteristics of the HPDM group scaffolds.

To further investigate the particle size, potential, and microscopic morphology of the degradation products, the scaffold degradation solution was centrifuged to remove the supernatant, resuspended in deionized water, and treated with an ultrasonic disperser (China, Shanghai) to ensure uniform dispersion of the precipitate. Take 1 mL of the sample and add it to the measurement cell. The particle size and potential of particles in the sample are detected using a nanoparticle size and Zeta potential analyzer (England, Malvern Panalytical). Take another 2 mL of the degradation solution, shake it thoroughly to mix well, then directly adhere the droplet to the front side of the copper grid. Quickly place the back side of the copper grid onto absorbent paper and let it stand for approximately 2 h. After allowing it to air-dry naturally, a transmission electron microscope (Japan, Hitachi) will capture the sample morphology. In addition, the scaffold samples after 8 weeks of dynamic degradation were taken for SEM observation.

### 2.4 Cell counting Kit-8 (CCK-8)

The cells used in this study include Raw264.7, MC3T3-E1, and HUVEC, which were purchased from the Chinese Academy of Sciences Cell Bank and Procell Biological Co., Ltd., respectively. To evaluate the cytotoxicity of three different scaffolds, cytotoxicity testing was performed using the CCK-8 kit (China, Bosterbio). Using a complete medium containing 10 % FBS (USA, Gibco) as the extraction medium, the scaffold was added to the medium at a volume ratio of 3 cm^2^/mL and soaked at 37 °C for 72 h. The third-generation cells were seeded at 5 × 10^3^ cells/well in a 96-well plate, with 200 μL of material extract added to each well. After 48 h of incubation (5 % CO_2_, 37 °C), the extract was aspirated and discarded, followed by washing the cells with PBS three times. Then, 10 μL of CCK-8 solution and 100 μL of fresh complete medium were added to each well, and the plate was incubated at 37 °C for 1 h. The absorbance at 450 nm was measured using a multifunctional microplate reader (USA, Thermo Scientific). All tests were repeated three times.

### 2.5 Live/dead cell staining

After high-temperature sterilization of the porous scaffold, it was placed in a 24-well plate. MC3T3-E1 and HUVECs were seeded at 2 × 10^4^ cells/well, respectively, followed by incubation with a complete medium for 48 h (5 % CO_2_, 37 °C). The medium was then aspirated and discarded, and the cells were gently washed with PBS three times. Add 1 mL of serum-free medium containing Calcein-AM/PI to each well, and incubate in the dark for 30 min (5 % CO_2_, 37 °C). Observe and photograph the staining results under a confocal laser scanning microscope (Japan, Olympus). All tests were repeated three times.

### 2.6 Hemolysis test

Collect blood from New Zealand rabbits, place it in an Erlenmeyer flask containing glass beads, and shake it for 10 min to remove fibrinogen. Then, add 10 times the volume of physiological saline and centrifuge (1500 r/min, 15 min), discard the supernatant, and repeat the process three times. Add pure water, PBS, and scaffold extract into separate EP tubes, respectively, then add 100 μL of erythrocyte suspension to each EP tube, followed by oscillating incubation for 1 h (37 °C, 60r/min). Observe the erythrocyte rupture situation, transfer 100 μL of supernatant to a 96-well plate, and measure the OD value at 540 nm wavelength using a multifunctional microplate reader. All tests were repeated three times.

### 2.7 Cell adhesion assay

Sterilize the porous scaffolds at high temperatures, then place them into 24-well plates according to grouping. Seed MC3T3-E1 and HUVEC cells at a density of 2 × 10^4^ cells/well and culture for 24 h (5 % CO_2_, 37 °C). After incubation, aspirate and discard the medium, wash three times with PBS, fix with 4 % paraformaldehyde for 30 min, treat with 0.5 % Triton X-100 for 15 min, and incubate with phalloidin staining solution (50 μg/mL) (China, Solarbio) at room temperature in the dark for 30 min. The cells were washed three times with PBS again, followed by staining with DAPI staining solution (China, Solarbio) for 15 min to visualize the nuclei. Cell adhesion was observed under a confocal laser scanning microscope to evaluate the adhesion properties of different scaffolds on MC3T3-E1 and HUVEC cells.

In addition, we fixed the scaffold materials cocultured with cells for 3 days with 2.5 % glutaraldehyde (China, Aladdin) for 3 h, followed by dehydration in 40 %, 50 %, 60 %, 70 %, 80 %, 90 %, 95 %, and 100 % ethanol solutions for 15 min each, and vacuum drying. The adhesion of cells on the surfaces of HP, HPD, and HPDM porous scaffolds was observed via SEM. All tests were repeated three times.

### 2.8 Electron paramagnetic resonance (EPR)

The hydroxyl radical scavenging capacity of HP, HPD, and HPDM porous scaffolds was detected using EPR (Bruker, Germany). First, it generates hydroxyl radicals using the TiO_2_/UV light (340 nm) system. Subsequently, place the porous scaffold into this mixed system and incubate for 60 min, then capture the remaining hydroxyl radicals with 5,5-Dimethyl-1-pyrroline N-Oxide (DMPO, China, Aladdin). EPR spectroscopy was used to evaluate the scavenging efficiency of scaffolds against hydroxyl radicals. All tests were repeated three times.

### 2.9 Detection of cellular reactive oxygen species (ROS)

2′,7′-Dichlorodihydrofluorescein diacetate (DCFH-DA) is a fluorescent probe for ROS detection. This study utilized the DCFH-DA detection kit (China, Solarbio) to measure the scavenging capacity of different porous scaffolds on intracellular ROS in Raw 264.7 cells. RAW 264.7 cells were seeded at a density of 1 × 10^4^ cells/well in the lower chamber of Transwell plates, incubated for 24 h, and then treated with 200 μM H_2_O_2_ for 12 h. Subsequently, the porous scaffold was placed in the upper chamber and cocultured for 12 h. A 10 μM DCFH-DA fluorescent probe was added to stain the cells for 30 min, followed by DAPI staining for 15 min. The intracellular ROS levels were detected using a fluorescence microscope (Japan, Olympus). All tests were repeated three times.

### 2.10 Immunofluorescence staining

To observe and compare the effects of different scaffolds on the expression of intracellular-related proteins after coculture with cells, the third-generation Raw 264.7, HUVEC, and MC3T3-E1 cells were cultured in confocal dishes with scaffold extracts added. After a specific incubation period, the medium was aspirated and discarded, and the cells were washed three times with PBS. The cells were then fixed with 4 % paraformaldehyde at room temperature for 30 min, washed three times with PBS, permeabilized with 0.1 % Triton X-100 for 15 min, and washed again with PBS. Blocking was performed using 10 % goat serum (China, Bosterbio) at room temperature for 1 h. Primary antibody incubation overnight (4 °C), fluorescent secondary antibody incubation at room temperature for 2 h, PBS washing of cells 3 times, DAPI nuclear staining for 15 min. Observe target protein expression under a confocal laser scanning microscope and quantitatively analyze target protein fluorescence intensity using Image J software. All tests were repeated three times.

### 2.11 Scratch test

Use a marker pen to draw three parallel lines on the outer bottom surface of a 6-well plate with a spacing of approximately 0.5 cm. Seed cells at a density of 2 × 10^4^ cells/well. When cells cover about 80 % of the bottom surface of the 6-well plate, aspirate and discard the culture medium. Use a 100 μL pipette tip to scratch parallel lines on the bottom surface vertically. Wash 3 times with PBS, then add serum-free medium. Place the cells back into the incubator to continue culturing (37 °C, 5 % CO_2_). Photographs were taken under a microscope at zero h, 12h, and 24h to observe cell migration. The width and area of the scratches at each time point were analyzed using Image J software. All tests were repeated three times.

### 2.12 Transwell assay

Coculture HUVEC or MC3T3-E1 cells (1 × 10^5^ cells/well) with various porous scaffolds in Transwell chambers, placing the scaffolds in the lower compartment and seeding the cells in the upper compartment. After 2 days of coculture, aspirate and discard the culture medium, wash 3 times with PBS, and carefully scrape off the cells on the front side of the Transwell chamber membrane using a cotton swab. The 4 % paraformaldehyde was used to fix the samples at room temperature for 30 min, then stained with crystal violet solution for 30 min. After washing with PBS again, the Transwell chamber membrane was gently removed with a scalpel and placed upside down on a glass slide. Observe the cell quantity and morphology under the microscope and perform quantitative analysis using Image J software. All tests were repeated three times.

### 2.13 Tube-forming experiment

Precool the culture medium, experimental consumables, and Matrigel matrix (USA, Corning Biotech) at 4 °C overnight. Dilute the Matrigel matrix with the serum-free medium at a 1:1 ratio, then add 100uL/well to a 24-well plate and incubate for 30 min until gelation occurs (37 °C). Subsequently, remove excess liquid from the surface of the Matrigel matrix. The third-generation HUVEC cells were seeded at 5 × 10^4^ cells/well in a 96-well plate, supplemented with different scaffold extracts, and incubated for 24 h (37 °C, 5 % CO_2_). Cell distribution morphology was dynamically observed under a microscope and photographed for documentation. Image-Pro Plus 6.0 software is used to perform lumen structure node scoring. All tests were repeated three times.

### 2.14 Alkaline phosphatase (ALP) staining

To simulate the impact of porous scaffolds on osteoblast differentiation within a multi-cellular coexistence environment in the body, we introduced an HPDM + “induction medium' group (HPDM@Hu/Ra). The specific preparation method for the “induction medium' is illustrated in Fig. S4, which involves collecting the supernatant after coculturing the HPDM porous scaffold with Raw264.7 and HUVEC cells for 24 h, followed by the addition of 10 % FBS to obtain the final product.

To investigate the effects of three types of porous scaffolds and HPDM@Hu/Ra on ALP expression in MC3T3-E1 cells. MC3T3-E1 cells were cocultured with different porous scaffolds at 1 × 10^5^ cells/well density in 24-well plates, with complete medium or “induction medium' added, respectively (37 °C, 5 % CO_2_). On the seventh day, the culture medium was aspirated and discarded, washed 3 times with PBS, stained using the ALP kit (China, Bosterbio), and observed under a microscope. All tests were repeated three times.

### 2.15 Alizarin red staining (ARS)

To evaluate the effects of three types of porous scaffolds and HPDM@Hu/Ra on the osteogenic mineralization capacity of MC3T3-E1 cells, the formation of calcium nodules in MC3T3-E1 cell specimens cultured for 21 days was assessed using an ARS kit (China, Bosterbio). MC3T3-E1 cells were cocultured with different porous scaffolds at 1 × 10^5^ cells/well density in complete medium or “induction medium' in 24-well plates (37 °C, 5 % CO_2_). Change the culture medium every 3 days. On day 21, aspirate and discard the medium, wash the cells 3 times with PBS, fix with 4 % paraformaldehyde for 30 min, stain with 1 % alizarin red solution for 30 min, and observe under the microscope after photographing. Quantitative analysis was performed using Image J software. All tests were repeated three times.

### 2.16 Genomic sequencing

The three types of cells were cocultured with porous scaffolds (MC3T3-E1 cells were additionally assigned to the HPDM@Hu/Ra group). Upon reaching the predetermined time points, the cells were lysed with TRIzol, cooled in liquid nitrogen for 5–10 min, and stored at −80 °C. The NanoDrop 2000 spectrophotometer was used to measure the purity and concentration of RNA, while the Agilent 2100/LabChip GX assessed RNA integrity. After passing sample quality control, library construction and quality control were performed. After passing the library quality inspection, high-throughput sequencing was performed using the Illumina NovaSeq6000 platform in paired-end 150bp (PE150) mode. To ensure data reliability, all tests were repeated three times. The sequencing data was analyzed through the BMK Cloud platform (www.biocloud.net).

### 2.17 Western-blot

After coculturing different cells with porous scaffolds, the culture medium was aspirated and discarded, then washed with PBS three times. RIPA lysis buffer (4 °C, 30 min) (China, Solarbio) was added to collect the lysate, which was then centrifuged for 10 min (12,000 rpm, 4 °C). The supernatant constituted the total protein solution. The protein concentration was measured using a BCA kit (China, Bosterbio), then protein loading buffer was added, followed by high-temperature denaturation (100 °C, 5 min), and stored at −80 °C for future use. Prepare SDS-PAGE gel (China, Epizyme Biotech), add protein samples and Marker, then perform electrophoresis (80V) and membrane transfer (200 mA). After the transfer is completed, remove and trim the PVDF membrane, rinse it with TBST buffer three times (5 min each time), block with protein blocking solution (60 r/min, 1 h), and then rinse again with TBST buffer five times (5 min each time). The target protein primary antibody (USA, Abcam) was thawed and diluted according to the manufacturer's instructions. Then, 2 mL of the diluted primary antibody solution was added to the antibody incubation chamber and incubated overnight (4 °C), followed by five washes with TBST buffer (10 min each). Add goat anti-rabbit secondary antibody solution, incubate at room temperature for 2 h, and rinse with TBST buffer. Apply chemiluminescent developing solution (China, Bosterbio) onto the PVDF membrane, then place it into the Chemi Doc XRS + detection system (USA, Thermo Scientific) for exposure. The grayscale values of the bands were analyzed using Image J software. All tests were repeated three times.

### 2.18 RT-qPCR

After coculturing various scaffolds with cells in 6-well plates for 3 or 7 days, aspirate and discard the medium, wash three times with PBS buffer, add 1 mL of Trizol (from China, Solarbio) to each well, let it stand on ice for 5 min, and then collect the lysate into a 1.5 mL enzyme-free EP tube. Add 200 μL of chloroform to each tube, mix thoroughly by pipetting, let stand on ice for 5 min, centrifuge for 15 min (4 °C, 14000 rpm), and observe the mixture separating into two layers. Then, transfer the supernatant to a new nuclease-free EP tube, add an equal volume of isopropanol, mix thoroughly by pipetting, let stand on ice for 10 min, and centrifuge for 10 min (4 °C, 14,000 rpm). After aspirating and discarding all the liquid, add 1 mL of 75 % ethanol solution, gently pipette to mix, and centrifuge for 10 min (4 °C, 7500g). Aspirate and discard the supernatant, then invert the EP tube on absorbent paper to allow it to air dry naturally. Then, add 20 μL of DEPC water to the EP tube to dissolve the RNA, and store the above samples in a −80 °C freezer for later use.

The RNA concentration and purity were measured using a micro-spectrophotometer (USA, Thermo Scientific). Following the reverse transcription kit instructions, RNA samples, nuclease-free water, and raw materials were sequentially added to perform reverse transcription, and the products were stored at −20 °C for later use. Subsequently, quantitative PCR reactions were performed using the instructions of the PCR kit (Japan, Takara Bio) and the pre-designed primer sequences (Table 1, Table 2, Table 3). Using GAPDH as the reference, the expression levels of the target genes were calculated using the normalized expression method (2^−ΔΔCt^) to determine the expression levels of the target genes. All tests were repeated three times.

**Table 1:** Primer sequences for real-time PCR studies (Raw264.7).

Name	Primer sequence
GAPDH	Forward: 5′-TGACCACAGTCCATGCCATC-3′
	Reverse: 5′-GACGGACACATTGGGGGTAG-3′
p65	Forward: 5′-ACTGCCGGGATGGCTACTAT-3′
	Reverse: 5′-TCTGGATTCGCTGGCTAATGG-3′
CD 86	Forward: 5′-CTGCTGGTTGCTGTGTTTGT-3′
	Reverse: 5′-GGGCTCTTCATCTTCACACG-3′
TNF	Forward: 5′-CTGAACTTCGGGGTGATCGG-3′
	Reverse: 5′-GGCTTGTCACTCGAATTTTGAGA-3′
IL-6	Forward: 5′-ATAGTCCTTCCTACCCCAATTTCC-3′
	Reverse: 5′-TGATGAATTGGATGGTCTTGGTCC-3′
IL-10	Forward: 5′-GAGAAGCATGGCCCAGAAATC-3′
	Reverse: 5′-GAGAAATCGATGACAGCGCC-3′
CD206	Forward: 5′-AGCTGACCCTGGTATGTCCT-3′
	Reverse: 5′-ATTGTCTTGAGGGGCTGGTG-3′
Arg-1	Forward: 5′-CATATCTGCCAAGGACATCG-3′
	Reverse: 5′-GGTCTCTTCCATCACTTTGC-3′
TGF-β1	Forward: 5′-CTCCCGTGGCTTCTAGTGC-3′
	Reverse: 5′-GCCTTAGTTTGGACAGGATCTG-3′

**Table 2:** Primer sequences for real-time PCR studies (HUVECs).

Name	Primer sequence
GAPDH	Forward: 5′-GGAGTCCACTGGCGTCTTC-3′
	Reverse: 5′-GCTGATGATCTTGAGGCTGTTG-3′
CD31	Forward: 5′-GACGTGCAGTACACGGAAGT-3′
	Reverse: 5′-GGAGCCTTCCGTTCTAGAGTAT-3′
VEGFA	Forward: 5′-AGTTCGAGGAAAGGGCAAGG-3′
	Reverse: 5′-CAGGGAACGCTCCAGGATTT-3′
EMCN	Forward: 5′-CTGGGAGAAGGTGGAGAAGG-3′
	Reverse: 5′-TCCAGGGTCTTGGTTGTCAT-3′
Notch 1	Forward: 5′-GCTACAACTGCGTGTGTGTC-3′
	Reverse: 5′-GTTGGTGTCGCAGTTGGAGC-3′
DLL 4	Forward: 5′-GGACCTGGAGAACCTGAACC-3′
	Reverse: 5′-TCCTTGTCCTTGGCTTTGTC-3′

**Table 3:** Primer sequences for real-time PCR studies (MC3T3-E1).

Name	Primer sequence
GAPDH	Forward: 5′-TGACCACAGTCCATGCCATC-3′
	Reverse: 5′-GACGGACACATTGGGGGTAG-3′
DLL 4	Forward: 5′-GAGGAGAGGAATGAATGTGTCA-3′
	Reverse: 5′-GCTGAGCAGGGATGTCCA-3′
RANKL	Forward: 5′-CCATCGGGTTCCCATAAAG-3′
	Reverse: 5′-TGAAGCAAATGTTGGCGTA-3′
OPG	Forward: 5′-TGAAAACATCCCACTTTCCCAAA-3′
	Reverse: 5′-AGCAGCTTATTTTCACGGATTGA-3′
BMP 2	Forward: 5′-TTGGACACCAGGTTAGTGAATCA-3′
	Reverse: 5′-TCTCCTCTAAATGGGCCACTT-3′

### 2.19 Animal experiment

#### 2.19.1 Regulation of macrophage polarization effects in vivo

All animal experiments in this study were reviewed and approved by the Laboratory Animal Ethics Committee of Chongqing Medical University (IACUC-CQMU-2022-0022). The experiment used an animal model of porous scaffold subcutaneous implantation in SD rats (Fig. S5). Eighteen 12-week-old male SD rats were purchased and randomly divided into three groups: HP, HPD, and HPDM (n = 6). After successful anesthesia with isoflurane, the surgical site was shaved and disinfected. A longitudinal incision (approximately 2 cm) was made along the midline of the rat's back. The skin and subcutaneous tissue were bluntly dissected, and porous scaffolds were implanted subcutaneously according to the experimental groups. The skin was then sutured layer by layer. On postoperative days 7 and 14, SD rats were euthanized with excessive anesthesia. Complete subcutaneous tissue specimens encapsulating the porous scaffold were collected and placed in tissue preservation solution (50 mL PBS + 10 mL FBS + 146.125 mg EDTA) for subsequent examination.

After fully mincing the soft tissue wrapped around the scaffold, transfer it into a 50 mL centrifuge tube. Add 5 mL of tissue digestion solution (10 mg collagenase I + 6 mg DNase I + 40 mL DMEM medium), shake for digestion for 4 h, filter (70 μm), centrifuge (300g, 10min) to collect cells, resuspend in cell preservation solution (49 mL PBS + 1 mL FBS), and perform cell counting. Approximately 600,000 cells were taken from each group and distributed into 6 EP tubes. The cells were fixed with 4 % paraformaldehyde for 30 min, followed by membrane permeabilization with 0.5 % Triton at room temperature for 15 min. Fluorescent antibodies (CD11b, CD86, CD206) (China, Proteintech Group, Inc) were used for staining, and the samples were incubated at room temperature in the dark for 30 min. After centrifugation, the cells were resuspended in PBS. Subsequently, detection was performed using a flow cytometer (Japan, Sony Biotechnology Inc.). All tests were repeated three times.

#### 2.19.2 Establishment of a bone defect model in vivo

Thirty-six male New Zealand rabbits were randomly divided into three groups, namely the HP, HPD, and HPDM groups (n = 6). The femoral condyle bone defect model was prepared according to the method reported in the literature [28,29]. The specific experimental procedures are as follows: Anesthetize the subject using an animal anesthesia machine (isoflurane). After successful anesthesia, shave the fur around the knee joint of the New Zealand rabbit and disinfect the surgical area with povidone-iodine. A skin incision (2 cm) parallel to the long axis of the femur was made along the lateral femoral condyle of the New Zealand rabbit. The skin and subcutaneous fascia were incised layer by layer to expose the lateral bone surface of the femoral condyle. Using a 6 mm drill bit with an electric bone drill, create a cylindrical bone defect approximately 10 mm deep in the lateral femoral condyle. According to the experimental grouping, implant the corresponding porous scaffold into the bone defect site (Fig. S6), rinse with saline, and suture the surgical incision layer by layer, followed by disinfection. Postoperatively, each New Zealand rabbit was intramuscularly administered 800,000 units of penicillin sodium per day for three consecutive days.

At the 6th and 12th weeks post-operation, New Zealand rabbits were euthanized using high-concentration carbon dioxide asphyxiation. The femoral condyle specimens were dissected and stored in 50 mL centrifuge tubes containing 4 % paraformaldehyde. A scalpel was used to expose the abdominal and thoracic cavities, examine the liver, kidneys, heart, and lungs, respectively, and collect tissue specimens fixed in 4 % paraformaldehyde for further examination. An electric saw was used to open the skull, excise a portion of brain tissue, and fix it for examination.

#### 2.19.3 Micro-CT

The soft tissues on the surface of the femoral condyle specimen were thoroughly cleaned, followed by scanning with Micro-CT (Germany, Bruker). The parameters were set: layer thickness 20 μm, voltage 70 kV, current 200 μA, exposure time 300 mS. The original images were imported into CT Analyser software for processing, with the scaffold implantation site selected as the region of interest (ROI) for three-dimensional reconstruction to observe the internal scaffold and surrounding newly formed trabecular bone. Quantitative analysis was simultaneously performed on the BMD, Tb. N, Tb. Th, and BV/TV of the region of interest.

At 6 weeks and 12 weeks after scaffold implantation, New Zealand rabbits were anesthetized, and their lower limb blood vessels were continuously perfused with heparin sodium saline (400 U/L) to wash out the blood thoroughly. Subsequently, 50 mL of Microfil MV-122 vascular perfusion agent (USA, Flow Tech, Inc.) was injected into the inferior vena cava. Upon successful perfusion, the lower limb blood vessels appeared yellow. The cadaver specimens were stored at 4 °C overnight, after which the femurs from both lower limbs were extracted and fixed in 4 % paraformaldehyde for 48 h. Decalcification was performed using 10 % EDTA for 4 weeks. A High-resolution micro-CT imaging system was employed to obtain vascular imaging within the bone defect area.

#### 2.19.4 Histological staining analysis

To observe the bone tissue ingrowth and calcification degree (maturity) inside the scaffold of femoral condyle specimens, hard tissue sectioning and HE, Goldner, TRAP, and immunohistochemical staining were performed on the femoral condyle specimens. Additionally, HE staining was conducted on visceral tissue specimens from New Zealand rabbits to evaluate the in vivo biocompatibility of the scaffold material.

#### 2.19.5 Bone dynamic histomorphometric analysis

Calcein labeling staining is a technique commonly used in the study of bone metabolism and dynamic histomorphometric analysis, enabling dynamic tracking of the bone formation process and reflecting the rate of bone formation. Ten days and three days before euthanizing the New Zealand rabbits, 10 mg/kg of calcein solution (China, Aladdin) should be injected intraperitoneally. The embedded specimens were sectioned using a hard tissue microtome, observed under a fluorescence microscope, and photographed. The mineral apposition rate (MAR) was measured and calculated using Image J software, with the calculation method as shown in Formula (2):

(2) MAR=[Markspacing()]/[Timeinterval(days)]

### 2.20 Statistical analysis

Statistical analysis was performed using SPSS 23.0 (IBM Corp., Armonk, NY, USA) and GraphPad Prism 9 (GraphPad Software, United States). Independent sample t-tests were used to assess statistical differences between the two groups, while one-way or two-way analysis of variance (ANOVA) was employed for multiple group comparisons. The data were calculated as the mean ± standard deviation (SD), and P < 0.05 was considered statistically significant.

## 3 Results

### 3.1 Porous scaffold synthesis and characterization

**Fig. 1:**
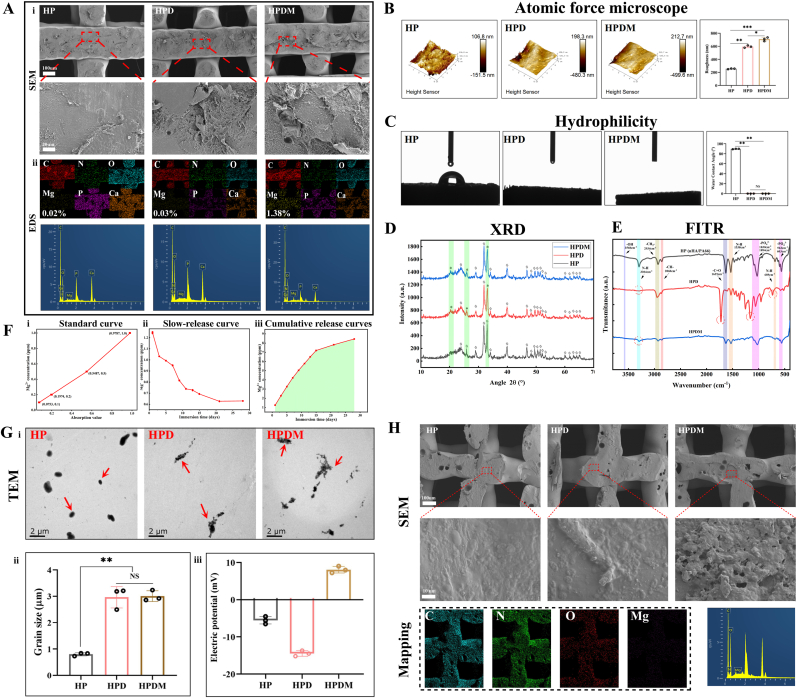
Characterization results of three types of porous scaffolds. (A) SEM observation of the porous scaffold's microscopic morphology and surface roughness, mapping scan of C, N, O, Mg, P, Ca element distribution, and quantitative analysis. (B) Further analysis of the surface roughness of the three porous scaffolds via AFM revealed that the HPDM scaffold exhibited the greatest height variation. (C) The water contact angle measurements of the three types of scaffolds showed that the HPD and HPDM scaffolds exhibited extremely high hydrophilicity, preventing water droplets from forming and aggregating on the scaffold surfaces. (D) XRD analysis of the crystal structures on the surfaces of HP, HPD, and HPDM scaffolds ("◇" marks the characteristic peaks consistent across all three scaffolds, "*" marks the characteristic peaks that differ among the three scaffolds, the green shaded area indicates the locations of the differential characteristic peaks). (E) FTIR detects the functional groups on the surfaces of HP, HPD, and HPDM scaffolds. The same color area represents identical absorption peaks, while the red dashed box indicates the locations of differential absorption peaks. (F) Characterization of Mg^2+^ release characteristics from HPDM scaffolds. i)The standard concentration curve of Mg^2+^ plotted according to the equipment characteristics; ii) The Mg^2+^ release concentration curve from days 1–28; iii) The cumulative concentration curve of Mg^2+^ from days 1–28. (G) The degradation products of three types of porous scaffolds include i) morphology, ii) particle size, and iii) zeta potential. (H) SEM observation of the morphological characteristics of three types of porous scaffolds after degradation, mapping scanning of the distribution of C, N, O, and Mg elements, and quantitative analysis of the Mg element. (n = 3, One-way ANOVA or Two-way ANOVA were used; *, p < 0.05; **, p < 0.01; ***, p < 0.001; NS, no significant difference). (For interpretation of the references to color in this figure legend, the reader is referred to the Web version of this article.)

The HP, HPD, and HPDM porous scaffolds exhibit a regular square pore structure with uniform pore size and good structural consistency. After modification with polydopamine, the surfaces of HPD and HPDM appear gray-black (Fig. S2). SEM-EDS scanning was used to observe the microstructure of the porous scaffolds. The surfaces of the HPDM and HPD scaffolds were rougher than those of the HP scaffold. C, N, O, P, and Ca were uniformly distributed on the surfaces of all scaffolds. The Mg content in the HPDM scaffold was 1.38 % (0.02 % in the HP scaffold and 0.03 % in the HPD scaffold), indicating that Mg^2+^ was successfully modified on the porous scaffolds (Fig. 1A).

The maximum height differences observed on the scaffold surface through AFM detection were HP ≈ 258.3 nm, HPD ≈678.6 nm, and HPDM ≈712.3 nm. After dopamine modification, the scaffold surface exhibits increased roughness, which is more conducive to cell adhesion (Fig. 1B). The hydrophilic test results showed that the water droplet structure on the surface of the HP scaffold remained intact with a relatively large contact angle. In contrast, water droplets were rapidly absorbed upon contact with the surfaces of the HPD and HPDM scaffolds, making it impossible to measure the water contact angle. This indicates a significant improvement in the hydrophilicity of both materials (Fig. 1C).

XRD test results indicate that the HP scaffold exhibits numerous sharp diffraction peaks, demonstrating that the scaffold material possesses good crystallinity. The diffraction peak patterns of HPD and HPDM scaffolds are consistent with those of the HP scaffold, with only minor differences in the intensity and width of some peaks, which may be related to the modification by polydopamine and Mg^2+^ (Fig. 1D). The FTIR analysis results show that compared to the HP scaffold, the HPD scaffold exhibits significant shifts in the -NH, -PO_4_^3-^, and -C=O stretching vibration peaks, and the N-H stretching vibration peak at 3304 cm^−1^ also disappears (Table 4). This may be related to the increased surface activity of the scaffold after polydopamine modification, which leads to changes in the elements' electron transfer and valence states. The characteristic peaks of the HPDM scaffold are consistent with those of the HP scaffold, but their intensity is somewhat diminished. This may be attributed to the alteration of the scaffold's overall negatively charged state following Mg^2+^ modification, as well as the “consumption' of dopamine during the reaction process, which allows the original functional groups of the HP scaffold to become “exposed' (Fig. 1E).

**Table 4:** Characteristic peaks for each functional group of nHA/PA66.

Functional group	nHA/PA66 Characteristic peak (cm^−1^)
-OH	621、3560
-PO_4_^3-^	562、603、1030、1094
N-H	3304
N-H	689、1538
-C=O	1639
-CH_2_-	2934
-CH-	2860

As shown in Fig. S10, from left to right are the nHA/PA66 porous scaffolds with HP, HPD, and HPDM pore sizes. The compressive mechanical properties of these three-dimensional porous scaffolds were tested. When the compression deformation displacement of the porous scaffold was 6 mm, all scaffolds were severely damage. At this point, the compressive modulus of the three scaffolds was similar.

### 3.2 Degradation analysis of porous scaffolds

The Mg^2+^ release behavior of HPDM scaffolds, including standard concentration curves, sustained-release curves, and cumulative release curves. The calculated Mg^2+^ loading rate of the HPDM scaffold was 69.32 ± 1.96 %. Meanwhile, the HPDM scaffold demonstrates favorable Mg^2+^ sustained-release properties, with the Mg^2+^ release rate gradually slowing over time and eventually stabilizing. The cumulative release curve indicates that the HPDM scaffold approaches saturation in Mg^2+^ release after 28 days, with an approximate cumulative release of 9 ppm (Fig. 1F).

The degradation products of the three scaffolds were observed using TEM. The degradation products of the HP scaffold were predominantly dot-like particles. In contrast, the HPD and HPDM scaffolds were mainly flocculent with small dot-like particles interspersed. The dot-shaped particles are nano-hydroxyapatite, while the flocculent material is polydopamine. The particle size and potential of the scaffold degradation products were analyzed using a Raman dynamic light scattering instrument. Among them, the degradation products of HP and HPD scaffolds exhibited negative charge potentials. In contrast, the degradation products of HPDM scaffolds showed positive charge potentials, which may be related to the presence of Mg^2+^ in the degradation products of HPDM scaffolds (Fig. 1G). Particle size analysis revealed that the degradation products of HP scaffolds had the smallest dimensions, while those of HPDM scaffolds exhibited the most significant dimensions.

The morphological characteristics of three types of scaffolds after two months of dynamic degradation were observed via SEM (Fig. 1H). All three types of scaffolds exhibit relatively uniform porous structures on their surfaces. Magnified images reveal that the HP scaffold surface appears smoother, whereas the HPD and HPDM scaffold surfaces display greater roughness. Energy spectrum analysis revealed a significant decrease in Mg content on the surface of the HPDM scaffold, indicating the gradual release of Mg elements during the degradation process.

### 3.3 Porous scaffold biocompatibility and cell adhesion

**Fig. 2:**
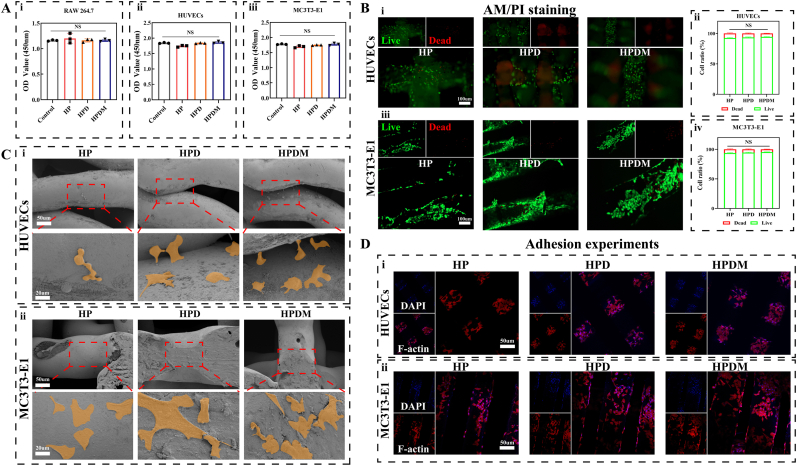
Biocompatibility of porous scaffolds. (A) CCK-8 results of RAW 264.7, HUVEC, and MC3T3-E1 cells cocultured with HP, HPD, and HPDM scaffold extracts (n = 5). (B) Analysis of live/dead staining for HUVEC (i & ii) and MC3T3-E1 (iii & iv) cells on different porous scaffold surfaces (n = 3). (C) Scanning electron micrographs of HUVEC (i) and MC3T3-E1 (ii) cells on different porous scaffold surfaces. (D) After 3 days of coculture, the cytoskeleton staining results of HUVEC (i) and MC3T3-E1 (ii) cells on different porous scaffold surfaces. (One-way ANOVA or Two-way ANOVA were used; NS, no significant difference).

The CCK-8 results indicate that the three scaffolds showed no significant cytotoxicity or inhibitory effects on the proliferation of RAW 264.7, HUVEC, and MC3T3-E1 cells. It can be concluded that the HP, HPD, and HPDM scaffolds exhibit good biocompatibility (Fig. 2A). It should be noted that the surface modification of nHA/PA66 porous scaffolds aims to enhance the adhesion and proliferation of HUVECs and MC3T3-E1 cells. Therefore, relevant experiments were only conducted for these two types of cells. As shown in Fig. 2B, HUVEC, and MC3T3-E1 cells exhibited favorable growth status on HP, HPD, and HPDM porous scaffolds, with a relatively high proportion of viable cells (abundant green fluorescent spots). All three porous scaffolds demonstrated good biocompatibility.

Hemolysis tests were conducted using extracts from three types of porous scaffolds with erythrocytes from New Zealand rabbits. The varying shades of color in the reaction system indicate different levels of red blood cell destruction, that is, the hemolytic effect (Fig. S7). The pure water group exhibited a deep red color, with red blood cells almost destroyed; the PBS, HP, HPD, and HPDM groups showed transparent colors, with red blood cells remaining largely intact. The results indicate that the three types of porous scaffolds exhibit good biocompatibility with blood and cause minimal damage to red blood cells, making them suitable for biomedical applications in vivo.

SEM observation of cell adhesion on the surface of three porous scaffolds revealed that cells exhibited the closest contact and most extensive cytoskeletal extension on the surfaces of HPD and HPDM porous scaffolds. In contrast, cell adhesion performance was relatively weaker on HP porous scaffolds (Fig. 2C). Further employing rhodamine-labeled phalloidin staining of HUVEC and MC3T3-E1 cytoskeletons to observe cell adhesion on the three porous scaffolds. It can be observed that cells are evenly distributed on all three types of porous scaffolds, exhibiting extended morphology, which indicates good cell adhesion on the scaffold surfaces. However, the HPD and HPDM porous scaffolds show higher cell density on their surfaces (Fig. 2D). Therefore, surface modification of HP porous scaffolds can enhance their cell adhesion properties.

### 3.4 HPDM scaffold regulates macrophage polarization and scavenges hydroxyl radicals in vitro

**Fig. 3:**
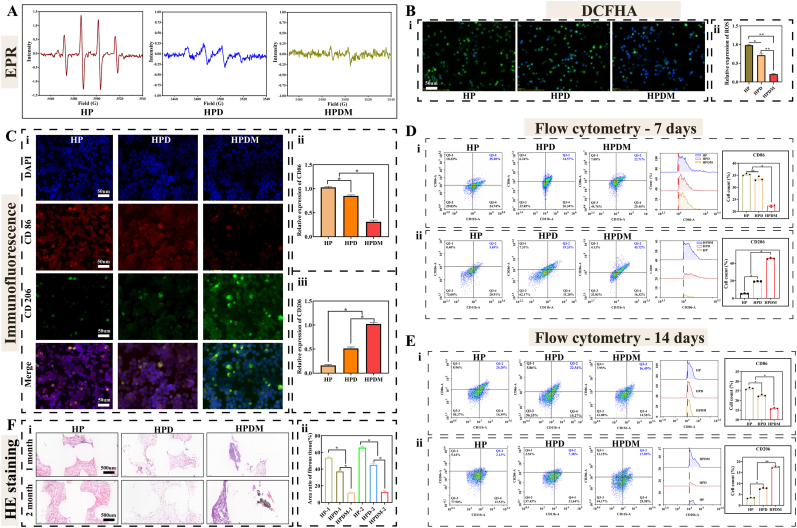
HPDM porous scaffold mediates immune regulation in vivo and in vitro. (A) EPR detects three porous scaffolds' -OH scavenging ability (HP, HPD, HPDM). (B) The DCFHA assay detects the ROS scavenging capacity of three porous scaffolds (HP, HPD, HPDM) (n = 3). (C) The immunofluorescence staining and quantitative analysis of CD86 and CD206 in macrophages cocultured with three types of porous scaffolds (HP, HPD, HPDM), respectively (n = 3). (D–E) Flow cytometry was utilized to detect macrophage polarization in the tissues surrounding the scaffold following 7 and 14 days of subcutaneous implantation (n = 3). (F) Analysis results of HE staining and area proportion of fibrous tissue in three types of porous scaffolds (HP, HPD, HPDM) (n = 6). (One-way ANOVA or Two-way ANOVA were used; *, p < 0.05; **, p < 0.01; NS, no significant difference).

The hydroxyl radical (-OH) scavenging capacity of three porous scaffolds was evaluated using EPR. As shown in Fig. 3A, the HP group exhibited characteristic -OH peaks (electron absorption peaks in a 1:2:2:1 ratio), whereas the -OH peaks were significantly attenuated in the HPD group and nearly disappeared in the HPDM group, indicating thorough elimination of -OH in the reaction system. The above results indicate that the HPDM scaffold possesses favorable -OH scavenging capability.

The DCFH-DA fluorescent probe was used to stain macrophages to evaluate the generation and clearance of intracellular reactive oxygen species (ROS). As can be seen from Fig. 3B, the HP group exhibits strong green fluorescence signals, indicating higher ROS generation. In contrast, the HPDM group shows significantly weakened green fluorescence signals, demonstrating better ROS scavenging capability.

The polarization regulation capability of three porous scaffolds on macrophages was observed through immunofluorescence staining. Fig. 3C shows that the HP group was predominantly polarized towards M1-type macrophages, characterized by high CD86 and low CD206 expression. The HPD group promoted M2-type macrophage polarization to some extent and reduced M1-type macrophage polarization, manifested as decreased CD86 expression and increased CD206 expression. However, the HPDM group significantly enhanced M2-type macrophage polarization, characterized by low CD86 and high CD206 expression.

### 3.5 HPDM scaffold regulates macrophage polarization and inhibits excessive fibroproliferation in vivo

At 7 days post-implantation of the porous scaffold into the subcutaneous tissue of SD rats, significant congestion was observed in the soft tissue surrounding the scaffold in the HP group, indicating a strong inflammatory response. The soft tissue surrounding the scaffolds in the HPD group exhibited a lighter color, indicating a reduced inflammatory response compared to the HP group. In contrast to the previous two groups, the HPDM group showed soft tissue coloration closest to normal with the mildest inflammatory reaction, demonstrating superior biocompatibility. At 14 days post-operation, inflammation in the peri-scaffold tissues of all three groups had subsided, but the HPDM group exhibited the best soft tissue healing around the scaffold (Fig. S8).

Further analysis of macrophage polarization in the soft tissues surrounding the subcutaneously implanted scaffolds was performed using flow cytometry. As shown in Fig. 3D, at 7 days post-operation, the HP group exhibited higher CD86 expression (35.6 ± 1.37 %) and lower CD206 expression in macrophages within the peri-implant soft tissues, indicating a higher proportion of M1 macrophages. Compared to the HP group, the HPD group showed no significant difference in macrophage CD86 expression but slightly higher CD206 expression. In contrast, the HPDM group demonstrated lower CD86 expression (21.9 ± 1.12 %) and significantly increased CD206 expression in macrophages. These results indicate that at 7 days post-subcutaneous implantation, the HPDM scaffold significantly promoted M2 macrophage polarization. At 14 days post-operation, the expression level of CD86 in macrophages within the soft tissue surrounding the scaffolds was HP > HPD > HPDM. Compared to the 7-day post-operation period, all groups showed a decrease, indicating a reduction in the proportion of M1-type macrophages. Additionally, the expression level of CD206 in macrophages within the soft tissue surrounding the porous scaffolds was HPDM > HPD > HP, and the CD206 expression at 14 days post-operation was lower than that at 7 days post-operation (Fig. 3E).

The HE staining results of porous scaffolds showed that the internal tissue of HP scaffolds exhibited significant inflammatory reactions at 7 days post-operation, with substantial fibrous tissue proliferation observed by 14 days post-operation. The internal tissue of HPD scaffolds displayed milder inflammatory reactions at 7 days post-operation, with abundant immature fibrous tissue within the scaffolds at 14 days post-operation. In contrast, the internal tissue of HPDM scaffolds demonstrated minimal inflammatory reactions and fibrous/scar tissue proliferation at both 7 and 14 days post-operation (Fig. 3F). It can be inferred that by postoperative day 14, the number of inflammatory cells gradually decreases as the local inflammation enters the chronic phase. At the same time, fibroblasts increase, leading to the proliferation of local fibrous tissue/scar tissue. Moreover, the HPDM scaffold performs optimally in suppressing inflammatory responses and excessive fibrotic proliferation.

### 3.6 HPDM scaffold promotes migration of HUVEC and MC3T3-E1 cells

**Fig. 4:**
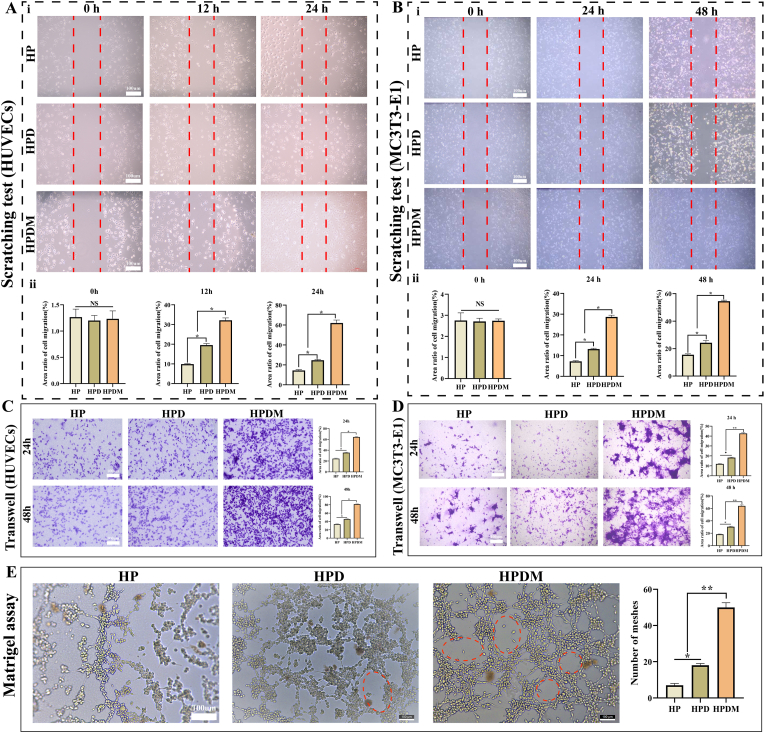
HPDM porous scaffold promotes cell migration and endothelial cell tube formation capability. (A)HUVEC cells, (B) MC3T3-E1 cells, and the scratch test results after co-culture with scaffold extracts. Results of transwell experiments after co-culture of three porous scaffolds with HUVEC cells (C) and MC3T3-E1 cells (D). (E) Results of three types of porous scaffolds inducing “tubular' formation in HUVEC cells. (n = 3, One-way ANOVA or Two-way ANOVA were used; *, p < 0.05; **, p < 0.01; NS, no significant difference).

Vascular reconstruction and bone tissue regeneration are inextricably linked. In the early stages of bone regeneration, endothelial cells must migrate to the bone defect area to reconstruct the local microcirculation, providing favorable conditions for material exchange during bone tissue regeneration. Therefore, we conducted Transwell and scratch assays to verify the effects of three types of porous scaffolds on the migration capabilities of vascular-forming and osteogenic cells. Scratch assay observations revealed that HUVEC cells gradually migrated over time into the scratched area. After 12 h of incubation, the cell migration area in the HPD and HPDM groups was significantly larger than in the HP group. After 24 h of incubation, the effect was even more pronounced in the HPDM group, indicating that the HPDM scaffold possesses a superior capability in promoting HUVEC cell migration (Fig. 4A). Similarly, after 24 h of incubation with MC3T3-E1 cells, the cell migration area in the HPD and HPDM groups was significantly higher than in the HP group. After 48 h of incubation, the cell migration area was even more pronounced in the HPDM group, indicating that the HPDM scaffold also exhibits superior capability in promoting MC3T3-E1 cell migration (Fig. 4B).

The Transwell experiment compares the effects of different porous scaffolds on cell migration ability by observing the number and distribution of cells traversing the polycarbonate membrane. As shown in Fig. 4C, after 24 h of coculture with three types of porous scaffolds, the HPDM group exhibited more migrated HUVEC cells than the HP and HPD groups. After 48 h of coculture, the number of migrated cells increased in all three groups, but the HPDM group still showed the highest cell migration count. This indicates that the HPDM scaffold performs best in promoting HUVEC cell migration. Like HUVEC cells, after 24 h of coculture with three types of porous scaffolds, the HPDM group exhibited the highest number of migrated MC3T3-E1 cells. After 48 h of coculture, all three groups increased the number of migrated cells. However, the HPDM group still showed the highest cell migration count, indicating that the HPDM scaffold most effectively promotes MC3T3-E1 cell migration (Fig. 4D). The above results indicate that the HPDM scaffold exhibits excellent efficacy in promoting the migration of both MC3T3-E1 and HUVEC cells, which may be related to the Mg^2+^ loaded on its surface.

### 3.7 HPDM porous scaffold promotes angiogenesis and osteogenic differentiation in vitro

The experimental results of tube formation demonstrated the influence of three types of porous scaffolds on the “tube-formation' capability of HUVEC cells. As shown in Fig. 4E, the HP and HPD groups exhibited fewer tubular structures, whereas the HPDM group displayed more tubular structures, indicating its strongest pro-angiogenic capability. The enhanced vascular formation promoted by the HPDM porous scaffold is associated with its surface-modified Mg^2+^.

**Fig. 5:**
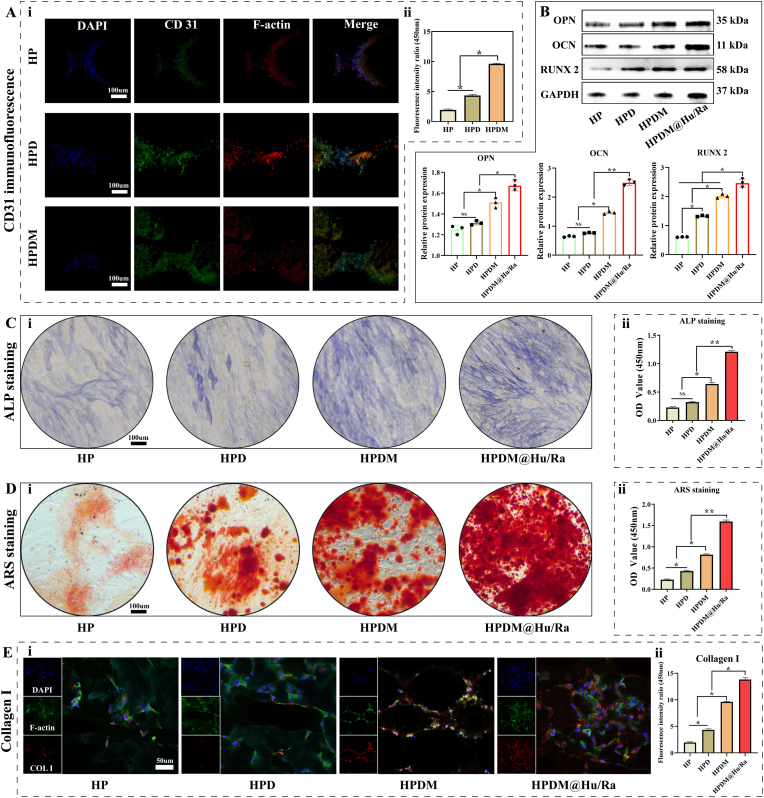
HPDM porous scaffold promotes vascular formation and osteogenic differentiation in vitro. (A) CD31 immunofluorescence staining in HUVEC cells. (B) Western blot detection and quantitative analysis of osteogenesis-related proteins OPN, OCN, and RUNX2 expression. (C) ALP staining and quantitative results. (D) ARS staining and quantitative results. (E) Immunofluorescence staining results of type I collagen in MC3T3-E1 cells. Group description: HPDM+ “induction medium' (HPDM@Hu/Ra). (n = 3, One-way ANOVA or Two-way ANOVA were used; *, p < 0.05; **, p < 0.01; NS, no significant difference).

In addition, we compared the pro-angiogenic effects of different scaffolds by observing the expression levels of angiogenesis-related protein (CD31) in HUVECs cocultured with three types of porous scaffolds through cellular immunofluorescence staining. As shown in Fig. 5A, HUVEC cells on the HPDM porous scaffold exhibited intense green fluorescence labeling. In contrast, the green fluorescence staining of HUVEC cells on the HPD and HP porous scaffolds was relatively weak. This indicates that the HPDM porous scaffold significantly promoted CD31 protein expression in HUVEC cells, demonstrating the optimal effect in facilitating vascular differentiation of HUVEC cells.

When validating the in vitro osteogenic differentiation-promoting effect of porous scaffolds, an HPDM + “induction medium' group (HPDM@ Hu/Ra) was added to simulate the influence of the in vivo environment on osteogenic regeneration. Thus, the in vitro osteogenic differentiation experiment included four groups: HP, HPD, HPDM, and HPDM@ Hu/Ra. Western blot results showed that the expression levels of osteogenic differentiation-related proteins OPN, OCN, and RUNX2 in the HPDM@Hu/Ra group were significantly increased compared to the HP, HPD, and HPDM groups (Fig. 5B). The ALP staining results showed that the HP and HPD groups exhibited the poorest staining effect, indicating lower ALP activity and insignificant osteogenic differentiation. The HPDM@Hu/Ra group demonstrated the most pronounced staining effect, reflecting the highest ALP activity and the most significant osteogenic differentiation. The HPDM group displayed a more noticeable staining effect than the HP and HPD groups, but it was inferior to the HPDM@Hu/Ra group (Fig. 5C). The ARS staining results showed that after 28 days of coculture between MC3T3-E1 cells and porous scaffolds, the HPDM@Hu/Ra group exhibited the most abundant red calcium nodules, indicating the highest degree of calcification deposition and mineralization. The HPDM group also demonstrated relatively high calcification deposition and mineralization levels, though inferior to the HPDM@Hu/Ra group. In contrast, the HPD and HP groups displayed weaker staining, with the least calcification deposition and lower mineralization levels (Fig. 5D).

Furthermore, the osteogenic differentiation effects of MC3T3-E1 cells under four different conditions were compared through COL I immunofluorescence staining. The results showed that the HPDM@Hu/Ra group exhibited the highest fluorescence intensity, indicating the strongest COL I expression in MC3T3-E1 cells under this condition. The HPDM group also displayed relatively high fluorescence intensity, while the HPD and HP groups showed weaker fluorescence intensity (Fig. 5E).

### 3.8 HPDM scaffold promotes vascularization and bone regeneration in New Zealand rabbits

At the 6th and 12th weeks post-operation, varying degrees of bone tissue regeneration were observed in New Zealand rabbits' femoral condyle bone defect sites. No fractures or pseudoarthrosis formation occurred in any of the femoral condyles. Micro-CT was utilized to evaluate newly formed bone tissue volume and skeletal parameters. At the same time, vascular perfusion was performed on the femoral condyle specimens to reconstruct the newly formed microvasculature in the bone defect area.

**Fig. 6:**
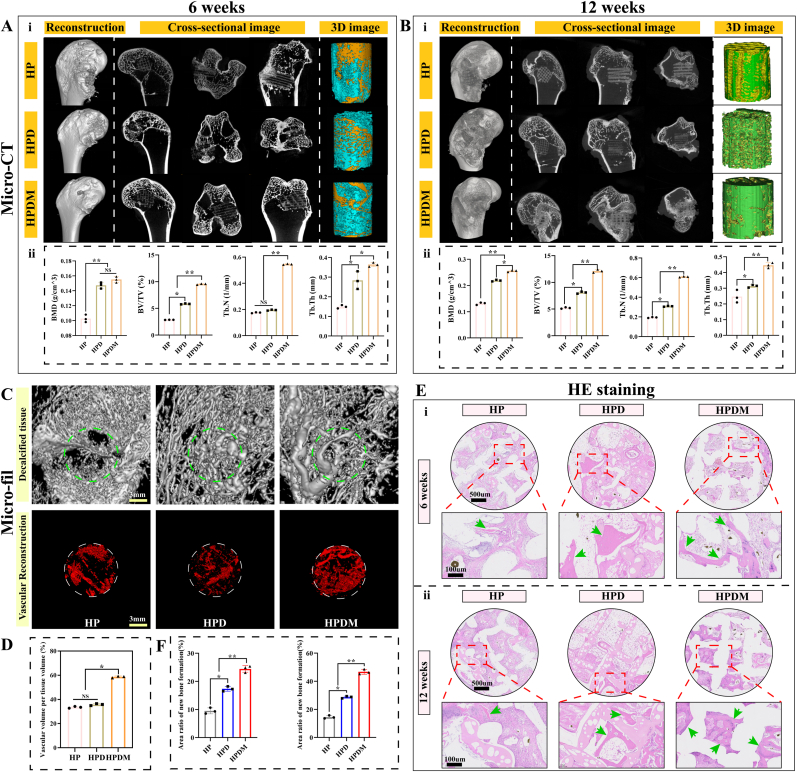
HPDM porous scaffold promotes vascular and bone regeneration in vivo. Coronal, transverse, sagittal, and CT reconstruction images of New Zealand rabbit femoral condyles at (A) 6 weeks post-operation and (B) 12 weeks post-operation, with newly formed bone tissue in the defect area marked in blue (6 weeks) and green (12 weeks), along with comparative analysis of bone parameters (BMD, BV/TV, Tb. N, Tb.Th). (C) CT reconstruction images of neovascularization and (D) volume comparison at the bone defect site in New Zealand rabbits 6 weeks post-operation. (E-F)Representative HE-stained images and quantitative analysis results at 6 and 12 weeks post-operation. The dashed box indicates the magnified image of the bone defect area, with green arrows pointing to the newly formed bone tissue. (n = 6, One-way ANOVA was used; *, p < 0.05; **, p < 0.01; NS, no significant difference). (For interpretation of the references to color in this figure legend, the reader is referred to the Web version of this article.)

Micro-CT results showed that at 6 weeks post-scaffold implantation, new bone tissue had grown into the scaffolds in the HP, HPD, and HPDM groups, with the HPDM scaffold group exhibiting the most significant growth (Fig. 6A). The HP scaffold demonstrated substantial bone tissue ingrowth around its periphery but minimal internal bone tissue, presenting a “hollow' appearance. In contrast, the HPDM group exhibited abundant bone tissue ingrowth around and inside the scaffold. Analysis of the newly formed bone tissue within the scaffold revealed that the HPDM group exhibited higher values than the HP and HPD groups in terms of bone mineral density (BMD), bone volume fraction (BV/TV), trabecular number (Tb.N), and trabecular thickness (Tb.Th). At 12 weeks post-scaffold implantation, the ingrowth of new bone tissue increased compared to that at 6 weeks post-operation, with the HPDM group still showing the most significant results (Fig. 6B). The newly formed bone tissue in the HP and HPD scaffold groups exhibited a sparse distribution of bone matrix and trabecular structure.

In contrast, the newly formed bone tissue in the HPDM scaffold group displayed a dense bone matrix and trabecular structure. Further analysis revealed that the HPDM scaffold group exhibited higher BMD, BV/TV, and Tb. Th, and Tb. N compared to the HP and HPD scaffold groups.

Furthermore, the micro-CT reconstruction results of blood vessels in newly formed bone tissue at 6 weeks post-implantation of porous scaffolds indicated that the HP group exhibited relatively fewer and sparsely distributed neovascular networks within the scaffolds. The HPD group demonstrated increased neovascularization with a slightly denser distribution than the HP group. Notably, the HPDM group displayed significantly enhanced neovascularization with markedly denser vascular networks within the scaffolds (Fig. 6C). A comparison of neovascularization volume among the three groups of porous scaffolds: The HP group scaffolds exhibited less neovascularization volume (approximately 30 %), the HPD group scaffolds showed a slight increase in neovascularization volume, but with no significant difference compared to the HP group, the HPDM group scaffolds demonstrated a significant increase in neovascularization volume (approximately 60 %), showing a significant difference compared to the HP and HPD groups (Fig. 6D). This indicates that the HPDM porous scaffolds possess superior pro-angiogenic effects.

**Fig. 7:**
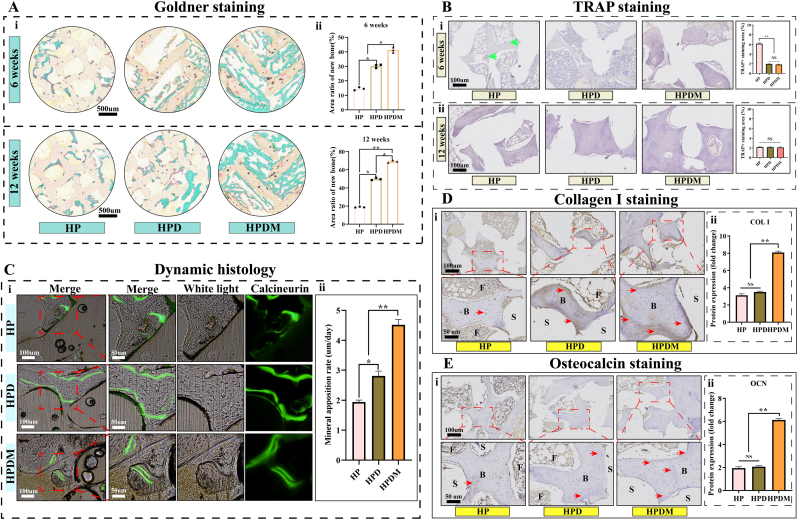
Histological analysis of new bone tissue in vivo. (A)At 6 and 12 weeks post-operation, Goldner staining and quantitative analysis of newly formed tissues inside the porous scaffold. (B)At 6 and 12 weeks post-operation, TRAP staining and quantitative analysis of new bone tissue inside the porous scaffold. (C)At 12 weeks post-operation, the calcein labeling produces new bone tissue within the porous scaffold. (D-E)Decalcified bone sections after immunohistochemical staining showed representative images of osteogenesis-related proteins COL I (6 weeks) and OCN (12 weeks), with measured expression differences of the related proteins. “B" represents bone tissue, “F" represents fibrous tissue, and “S" represents scaffold material. (n = 6, One-way ANOVA was used; *, p < 0.05; **, p < 0.01; NS, no significant difference).

To accurately evaluate the regeneration of bone tissue within the scaffold, further histological staining was performed to determine the type (bone matrix, cartilage, or fibrous tissue) and volume of newly formed tissue inside the scaffold. H&E staining results revealed that at 6 weeks post-implantation of the porous scaffold, the HP group exhibited predominantly fibrous hyperplasia with minimal bone tissue ingrowth. In the HPD group, proliferative fibrous tissue was reduced compared to the HP group, while newly formed bone tissue increased. However, the HPDM group demonstrated significantly diminished fibrous hyperplasia and a marked augmentation of newly formed bone tissue. At 12 weeks post-operation, the newly formed bone tissue within all three types of porous scaffolds had increased compared to 6 weeks post-operation. HPDM showed the most significant enhancement and exhibited almost no proliferative fibrous tissue. Therefore, HE staining results demonstrated that the HPDM scaffold could significantly promote bone regeneration while inhibiting fibrous tissue ingrowth (Fig. 6E). It should be noted that when preparing bone tissue sections, the sample must be cut into specimens no thicker than 5 μm. This process results in the partial detachment of the scaffold material within the bone tissue after it is severed. Goldner staining results revealed that at the sixth week post-scaffold implantation, the HPDM group exhibited more newly formed bone tissue (green areas) within the scaffold compared to the HP and HPD groups while displaying less newly formed fibrous tissue (light red areas) inside the scaffold relative to the HP and HPD groups (Fig. 7A). At 12 weeks post-operation, the HPDM group exhibited significantly increased new bone tissue formation within the scaffold. In contrast, the HP and HPD groups showed relatively less new bone tissue growth inside the scaffolds. Quantitative analysis of the volumetric proportion of newly formed bone tissue within the scaffolds revealed that the HPDM group demonstrated a markedly higher percentage of new bone tissue area than the other two groups at the 6-week and 12-week postoperative time points.

Tartrate-resistant acid phosphatase (TRAP) is an enzyme highly expressed in osteoclasts, commonly used to detect osteoclast quantity and activity. TRAP staining was performed on bone tissue specimens at 6 and 12 weeks post-operation to clarify further the metabolic status of newly formed bone tissue within the scaffold. As shown in Fig. 7B, at 6 weeks post-operation, a small number of TRAP + cells were present in the HP group. In contrast, no TRAP + cells were observed in the HPD and HPDM groups, indicating that polydopamine and Mg^2+^ exhibit inhibitory effects on osteoclast activity. At 12 weeks post-operation, the number of TRAP + cells in the HP group significantly decreased compared to 6 weeks post-operation, while no TRAP + cells were observed in the remaining two groups. The TRAP staining results indicated several osteoclasts were present during the early stage of HP scaffold implantation. The HPDM scaffold, modified with Mg^2+^ and polydopamine, significantly reduced the number of osteoclasts.

To investigate whether there are differences in the rate of bone tissue growth among different scaffold groups, a dynamic analysis of newly formed bone tissue was conducted using calcein labeling at 12 weeks post-scaffold implantation. Undecalcified bone tissue sections were prepared, and the distance between calcein labeling lines was observed under a fluorescence microscope to calculate the mineral apposition rate (MAR). The results indicate that the MAR in the HPDM scaffold group was significantly higher than in the HPD and HP scaffold groups (Fig. 7C). The above findings may be attributed to promoting vascular regeneration and bone metabolism within the scaffold by Mg^2+^, leading to a more rapid late-stage bone formation process.

COL I and OCN are early and late-stage expression products during the differentiation and maturation of bone tissue, respectively. Therefore, COL I staining was performed on the newly formed bone tissue within the scaffold 6 weeks post-operation. In comparison, OCN staining was conducted 12 weeks post-operation to assess the degree of bone tissue differentiation and calcification. The analysis results of COL I expression levels in the newly formed bone tissue within the three groups of scaffolds showed that the HP group exhibited lower COL I expression. In contrast, the HPD group demonstrated increased COL I expression, though without significant difference compared to the HP group.

In contrast, the HPDM group displayed significantly elevated COL I expression. These findings indicate that the HPDM porous scaffold can enhance COL I expression in bone tissue, promoting early-stage bone tissue differentiation (Fig. 7D). At 12 weeks post-operation, the analysis results of OCN expression levels in new bone tissue within the scaffolds across the three groups showed that the HP group exhibited lower OCN expression, and the HPD group demonstrated no significant increase in OCN expression. In contrast, the HPDM group displayed a significant elevation in OCN expression. This indicates that the HPDM porous scaffold can markedly enhance OCN expression in bone tissue, promoting the calcification and maturation of new bone tissue (Fig. 7E).

### 3.9 The HPDM scaffold inhibits excessive inflammation by regulating macrophage polarization

**Fig. 8:**
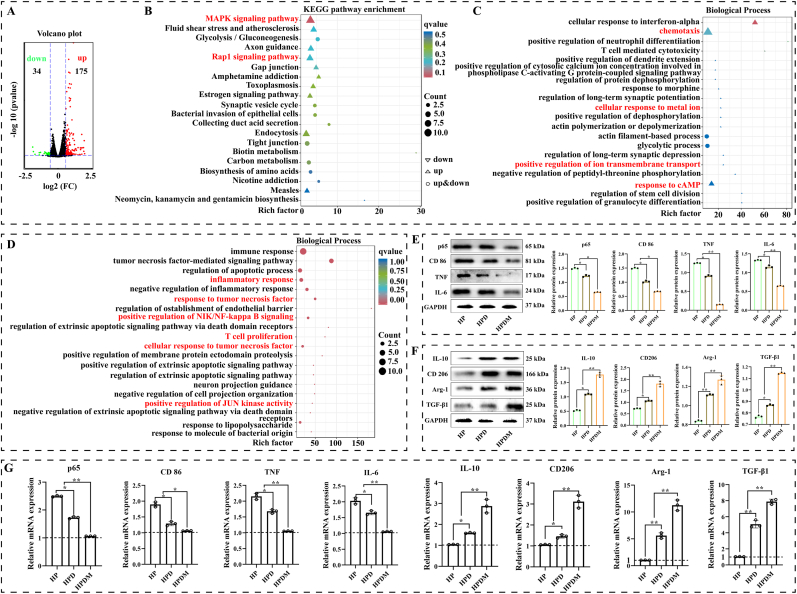
HPDM scaffold regulates macrophage polarization to suppress excessive inflammation. Transcriptome sequencing of Raw264.7 cells, (A) Volcano plot analysis showing the number of upregulated/down-regulated differentially expressed genes. KEGG enrichment analysis (B) and GO enrichment analysis (C-D) were performed on all differentially expressed genes, and GO enrichment analysis specifically targeted down-regulated differentially expressed genes. (E-F)Western blot validated the protein expression levels of pro-inflammatory and anti-inflammatory factors. (G)RT-qPCR validation of mRNA expression levels of pro-inflammatory and anti-inflammatory factors. (n = 3, One-way ANOVA was used; *, p < 0.05; **, p < 0.01; NS, no significant difference).

To elucidate the mechanism by which HPDM scaffolds regulate macrophage polarization and suppress excessive inflammatory responses, we cocultured HP and HPDM scaffolds with Raw 264.7 cells for 3 days, followed by RNA sequencing. Analysis of differentially expressed genes revealed that the HPDM group exhibited 209 differentially expressed genes compared to the HP group, including 175 upregulated genes and 34 downregulated genes (Fig. 8A). Further, KEGG and GO enrichment analyses were performed on all differentially expressed genes. Significant changes were observed in the expression of “MAPK, Rap1 signaling pathway' and biological processes such as “chemotaxis, cellular response to metal ion, response to cAMP' (Fig. 8B and C). Based on this, GO enrichment analysis of the 34 downregulated genes in the HPDM group revealed significant suppression in aspects such as “inflammatory response, TNF/NF-κB signaling pathway' (Fig. 8D).

Western blot and qPCR were further employed to validate the ability of HPDM scaffolds to induce macrophage differentiation into M1/M2 phenotypes. The specific protein and nucleic acid expression levels of M1 cell markers (CD86) along with their pro-inflammatory factors (p65, TNF, and IL-6), as well as M2 cell markers (CD206) and their anti-inflammatory factors (IL-10, TGF-β1, Arg-1), were quantitatively analyzed. The results demonstrated significant suppression in the expression of p65 (an activator of the NF-κB signaling pathway), CD86, TNF, and IL-6, while the expression levels of CD206, IL-10, TGF-β1, and Arg-1 were markedly upregulated (Fig. 8E–G).

### 3.10 The mechanism by which HPDM scaffolds promote vascular differentiation of HUVEC cells

**Fig. 9:**
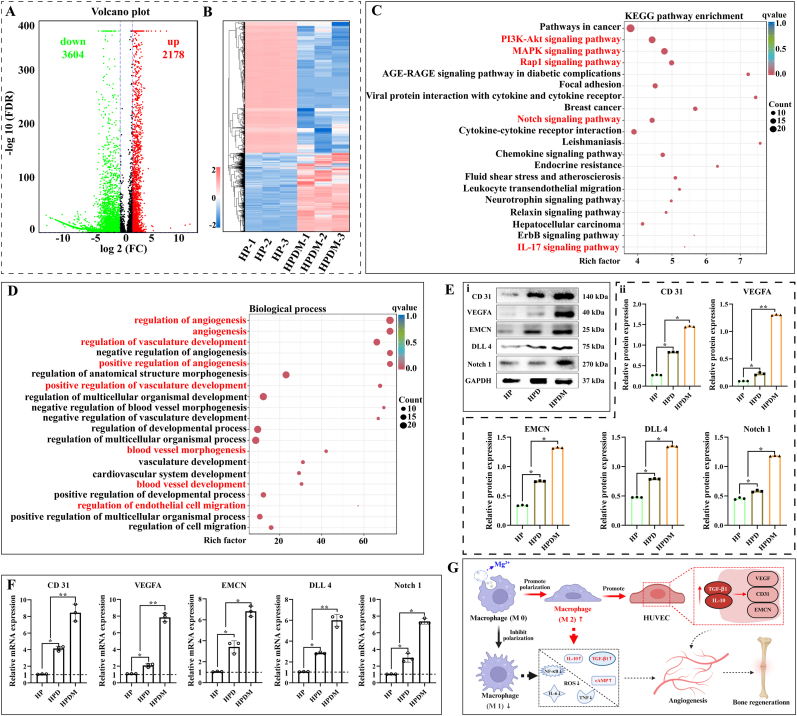
HPDM scaffold modulates macrophage polarization to promote vascular regeneration. (A) Transcriptome sequencing of HUVEC cells, volcano plot analysis showing the number of upregulated/downregulated differentially expressed genes, (B) followed by cluster analysis of all differentially expressed genes. KEGG enrichment analysis (C) and GO enrichment analysis (D) were performed on the upregulated differentially expressed genes. (E)Western blot and (F) RT-qPCR were used to validate protein and mRNA expression levels of angiogenesis-related factors (H-type vessels). (G) The mechanism of HPDM scaffolds regulates macrophage polarization to promote vascular regeneration. (n = 3, One-way ANOVA was used; *, p < 0.05; **, p < 0.01; NS, no significant difference).

To determine whether coculturing HUVEC cells with HPDM scaffolds can activate angiogenesis-related factors/signaling pathways and promote vascular formation, sequencing analysis was performed on HUVEC cells cocultured with HP and HPDM scaffolds, respectively. The results showed that compared to the HP group, the HPDM group exhibited 2178 upregulated genes and 3604 downregulated genes (Fig. 9A and B). KEGG enrichment analysis of the 2178 upregulated genes revealed that signaling pathways related to inflammation and angiogenesis, such as PI3K-Akt, MAPK, Rap1, Notch, and IL-17, ranked among the top 20. Furthermore, GO enrichment analysis also demonstrated that the HPDM scaffold holds significant implications in promoting endothelial cell migration, regulating vascular endothelial morphology, and facilitating vascularization (Fig. 9C and D). These results preliminarily confirm the capability of the HPDM scaffold to promote vascular differentiation in HUVEC cells.

Western blot and qPCR were employed to detect angiogenesis-specific proteins and mRNA expression levels in HUVEC cells cultured on the HPDM scaffold to ensure the accuracy of transcriptome gene sequencing results. As shown in Fig. 9E–F, the expression levels of “H"-type vessel-related proteins and nucleic acids such as CD31, VEGFA, and EMCN were significantly upregulated in the HPDM scaffold group. Moreover, the expression levels of Notch signaling pathway-related factors Notch 1 and DLL 4 in HUVEC cells were also markedly upregulated after intervention with the HPDM scaffold, and the Notch signaling pathway is a key regulatory pathway for vascularized osteogenesis (Fig. 9G).

### 3.11 The mechanism by which HPDM scaffolds promote osteogenic differentiation of MC3T3-E1 cells

**Fig. 10:**
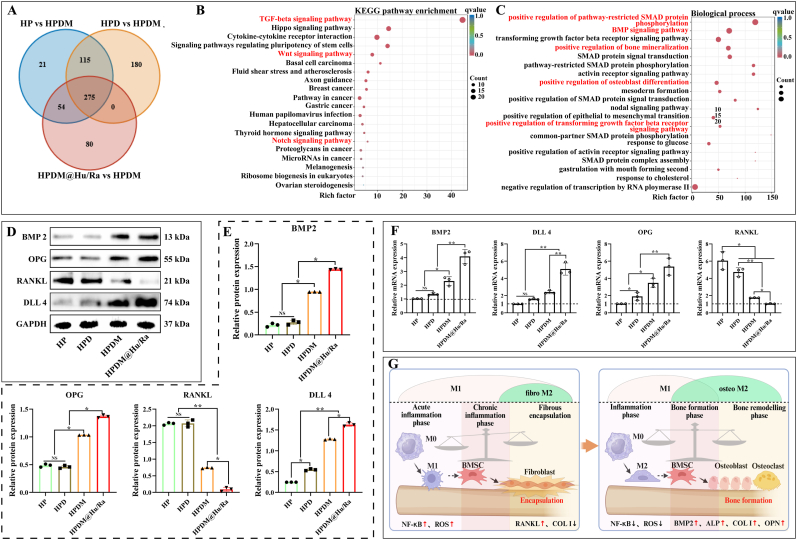
HPDM scaffold promotes vascularized bone regeneration through immune regulation. Transcriptome sequencing of MC3T3-E1 cells, (A) Venn analysis of differentially expressed genes, and KEGG analysis (B) and GO enrichment analysis (C) of 275 co-expressed differentially expressed genes. (D–E) Western blot and (F) RT-qPCR validated osteogenesis and bone metabolism-related factors' protein and mRNA expression levels. (G) The mechanism by which HPDM scaffolds promote vascularized bone regeneration through immune regulation. (n = 3, One-way ANOVA was used; *, p < 0.05; **, p < 0.01; NS, no significant difference).

To elucidate the mechanism by which the HPDM scaffold promotes osteogenic differentiation of MC3T3-E1 cells, transcriptome sequencing analysis was performed to assess the effects of three porous scaffolds on the gene expression of MC3T3-E1 cells, with comparisons made to the HPDM@Hu/Ra group. The results showed that the HPDM@Hu/Ra group had 275 co-upregulated genes compared to the HPDM group, HPD group, and HP group (Fig. 10A). KEGG enrichment analysis of these 275 genes revealed that the TGF-β signaling, Wnt signaling, and Notch signaling pathways ranked 1st, 5th, and 15th, respectively (Fig. 10B). GO enrichment analysis demonstrated significant upregulation in the biological process category for p-SMAD, BMP signaling pathway, positive regulation of osteoblast differentiation, and positive regulation of TGF-β receptor signaling pathway (Fig. 10C). These findings indicate that the HPDM scaffold is crucial in promoting bone formation. At the same time, the combined involvement of macrophages and endothelial cells further enhances vascularization and osteogenesis, which may be closely associated with interactions among multiple cell types.

To further confirm the effect of the HPDM scaffold combined with “induction medium' on the osteogenic differentiation of MC3T3-E1 cells, Western blot, and qPCR were used to detect the expression of osteogenic differentiation and bone metabolism-related proteins in MC3T3-E1 cells under different culture conditions. As shown in Fig. 5B, compared with the HP, HPD, and HPDM groups, the expression levels of RUNX2, OPN, and OCN in the HPDM@Hu/Ra group were significantly upregulated. Meanwhile, the expression of RANKL in the HPDM@Hu/Ra group was significantly downregulated, while osteoprotegerin (OPG) was significantly upregulated. In addition, the expression levels of osteogenesis-related signaling molecules (BMP 2; DLL 4) in the BMP signaling pathway and Notch signaling pathway were also significantly upregulated (Fig. 10D–F). The above results indicate that intervention with HPDM scaffolds on macrophages can suppress local excessive inflammation, remodel the M1/M2 balance, prevent fibrous encapsulation at the bone defect site, and promote pre-osteoblast differentiation toward osteogenesis (Fig. 10G).

### 3.12 Biocompatibility in vivo

At 12 weeks post-operation, New Zealand rabbits from each group were euthanized. Dissection of the abdominal cavity revealed no significant effusion or hemorrhage, with no adhesions or necrosis observed in the abdominal organs. HE staining of liver and kidney tissue sections showed no apparent abnormalities. Examination of the thoracic cavity demonstrated well-inflated lung tissue, intact pericardial structure, and normal cardiac position and morphology, with HE staining of tissue sections revealing no significant abnormalities. Upon opening the skull, the cerebrospinal fluid appeared clear, and the brain tissue morphology remained intact, with HE staining showing no cerebral infarction foci (Fig. S9).

## 4 Discussion

The number of patients worldwide requiring bone grafting due to critical-sized bone defects continues to rise annually. Autologous bone grafting and allogeneic bone grafting currently serve as the “gold standard' treatment methods. However, they are constrained by limitations such as restricted sources and disease transmission, creating an urgent need to explore new bone graft substitutes. nHA/PA66 is a novel biomaterial widely applied in the orthopedic field in recent years. Its composition and structure resemble the bone matrix, exhibiting excellent biocompatibility, mechanical properties, osteoinductivity, and osteoconductivity [30,31]. Like most hard tissue engineering materials, nHA/PA66 exhibits high strength and hardness, making it challenging to process precisely to meet the therapeutic requirements for critical-sized bone defects. In this study, we utilized the freeform 300-3X printer from ARBURG and APF technology to successfully fabricate nHA/PA66 porous scaffolds with millimeter-scale micropores. Functional modifications addressed complex cell migration within the scaffold, poor vascularization capability, insufficient osteoinductive quality, and excessive fibrotic invasion.

In recent years, polydopamine has been widely applied in the functional modification of biomaterial surfaces, and numerous studies have reported that Mg^2+^ exhibits favorable biological performance in promoting osteogenesis [32,33] and angiogenesis [34]. This study utilized the coordination reaction between polydopamine and Mg^2+^ to modify nHA/PA66 porous scaffolds with a pore size of 400 μm, resulting in functionalized scaffolds named nHA/PA66-DOPA-Mg (HPDM) that exhibit anti-inflammatory, angiogenic, and osteogenic effects. The modified HPDM scaffold was characterized using SEM, FTIR, and water contact angle measurements, demonstrating its surface roughening, excellent hydrophilicity, and high stability. The hydrophilicity of bone implant materials is a crucial factor influencing osseointegration capability. For porous implants, enhancing material hydrophilicity can promote cell adhesion and differentiation [35], improve bone matrix attachment on the implant surface [36], and accelerate mineral deposition on the material surface [37]. Good biocompatibility is a prerequisite for the use of biomaterials. The CCK8 experimental results indicate that the magnesium ions released by the HPDM scaffold also promoted the proliferation of MC3T3-E1 cells and HUVECs, consistent with the findings reported in the literature [38]. In addition, the cell adhesion of the modified scaffold was verified, and the results showed that polydopamine and Mg^2+^ could improve the cell adhesion on the surface of the porous scaffold.

Meanwhile, in vitro experiments also verified the degradation process and immunomodulatory effects of different types of scaffolds and their coatings. The results demonstrated that polydopamine and Mg^2+^ can effectively scavenge hydroxyl radicals and ROS while regulating macrophage polarization effects. The rat subcutaneous embedding experiment further verified that the HPDM porous scaffold exhibits excellent immune regulation effects in vivo.

Angiogenesis is crucial for bone repair, as newly formed blood vessels are the foundation for material exchange. This study analyzed the effects of different scaffolds on the angiogenic differentiation of HUVECs through scratch assays and tube formation experiments. The results demonstrated that the HPDM scaffold significantly enhanced the migration capability and tube formation of HUVECs. According to reports, H-type blood vessels can regulate the spatiotemporal coupling of angiogenesis and bone regeneration [39]. In this study, the CD31 protein expression level in the HPDM scaffold group was significantly higher than in the HP and HPD scaffold groups, suggesting that the HPDM scaffold can promote type H vessel formation, which may be related to Mg^2+^ [40]. The impact of bone repair implants on cellular osteogenic differentiation is crucial in determining bone regeneration. COL I and ALP are typical protein products during osteoblast differentiation and extracellular matrix maturation, often used to reflect the extent of early osteoblast differentiation [41]. COL I and ALP staining results showed that the HPDM scaffold group could significantly promote early osteoblast differentiation. ARS staining and OCN protein quantitative detection results demonstrated that after coculturing MC3T3-E1 cells with the HPDM scaffold, the extracellular matrix mineralization and maturation capacity was markedly enhanced. The above experimental results demonstrate that polydopamine and Mg^2+^ surface modification significantly enhance the cell adhesion, cell migration, angiogenesis, and osteogenic differentiation capabilities of nHA/PA66 porous scaffolds while inhibiting fibrosis invasion caused by excessive inflammation.

In vivo, experiments can more effectively evaluate the bone defect repair effects of different porous scaffolds. This study used New Zealand rabbits as experimental subjects to compare vascularization and bone ingrowth within the scaffolds at different time points after implantation. Micro-CT results showed that the HPDM group exhibited the most significant vascular growth within the scaffold at 6 weeks post-operation, which was markedly higher than that in the HP and HPD groups. Combined with the Mg^2+^ release characteristics of the HPDM scaffold and its pro-angiogenic differentiation effect on HUVECs in vitro, it can be inferred that the HPDM scaffold promotes vascular growth within the porous scaffold through the release of Mg^2+^. Furthermore, both micro-CT and bone tissue sections demonstrated significantly greater bone ingrowth within the scaffolds of the HPDM group compared to the other two groups at both 6 and 12 weeks postoperatively. It should be noted that the scaffold structure is prone to damage due to the thickness of stiff tissue sections, leading to the false appearance of “inconsistent' pore sizes. Meanwhile, histological sections only reflect the differences in bone ingrowth at a single cross-sectional level, making their results less accurate than Micro-CT. However, both micro-CT and bone tissue histological sections' experimental results demonstrate that the HPDM group scaffolds exhibit greater bone ingrowth volume, indicating that polydopamine and Mg^2+^ surface modification significantly enhance the osteogenic capacity of nHA/PA66 porous scaffolds.

Macrophages are an important component of immune cells, participating in the inflammatory response and repair process following bone defects [42]. Macrophages are a highly heterogeneous type of immune cells that typically polarize into M1 or M2 phenotypes under the stimulation of local microenvironmental cues [43]. M1 cells secrete pro-inflammatory factors such as IL-6, exacerbating local oxidative stress and inflammatory responses at bone trauma sites, thereby impeding bone repair [44]. M2 cells secrete anti-inflammatory factors such as TGF-β and IL-10, which suppress local inflammatory responses, recruit mesenchymal stem cells and vascular endothelial cells to the bone defect area, initiate the vascularization process [45,46], and promote bone repair. After the porous bone repair scaffold is implanted, M1 macrophages release inflammatory factors to recruit immune cells, triggering a cascade of inflammatory responses that may develop into chronic inflammation. This is one of the leading causes of bone defect repair failure [47]. Therefore, timely regulation of macrophage polarization toward the M2 phenotype is crucial for the success of bone regeneration. This study's in vivo experimental results demonstrate that the HP scaffold exhibited a higher proportion of M1 cell polarization during the early inflammatory phase. However, the HPDM scaffold implantation did not induce excessive M1 polarization in the early inflammatory stage while promoting a significant increase in M2 cells. This mechanism helps suppress excessive inflammatory responses during the early and middle stages of bone repair, thereby facilitating the establishment of an immune microenvironment conducive to bone regeneration at an earlier phase.

This study found that the HPDM scaffold regulates macrophage polarization and possesses properties that promote angiogenesis. Bone microvasculature provides oxygen, nutrients, and growth factors and transports osteogenic precursor cells during the bone repair process, playing a crucial role in bone regeneration [48,49]. For example, VEGFA can activate VEGFA receptors on osteoblasts to promote bone regeneration. WB and qPCR results showed that the HPDM group significantly promoted VEGFA expression in HUVEC cells, indicating that Mg^2+^ and polydopamine facilitate macrophage polarization toward the M2 phenotype and indirectly enhance angiogenesis. This demonstrates that angiogenesis and immune responses are interdependent. M2 macrophages also secrete cytokines involved in angiogenesis (such as TGF-β) to assist in blood vessel formation [50]. Furthermore, the increased expression of BMP-2 and TGF-β can indirectly promote angiogenesis and osteogenic differentiation through paracrine mechanisms. In this study, a significant elevation in BMP-2 and TGF-β expression levels was also observed in the HPDM group, indicating that Mg^2+^ synergized with polydopamine promotes macrophage polarization toward M2 phenotype to enhance angiogenesis and osteogenic differentiation.

## 5 Conclusion

nHA/PA66 is a commonly used bioactive material in orthopedics to expand its clinical application scope—treating critical-sized bone defects. This study successfully achieved 3D printing of nHA/PA66 porous scaffolds through APF technology. It modified the scaffolds using polydopamine and Mg^2+^ coordination reactions, obtaining HPDM porous scaffolds with surface roughening, high hydrophilicity, excellent adhesion, and superior biosafety. The HPDM porous scaffold exhibits excellent local immune regulation and ROS scavenging capabilities both in vivo and in vitro, as well as the ability to suppress local chronic inflammation and fibrous tissue invasion within the scaffold, thereby achieving vascularized bone regeneration inside the scaffold. The specific mechanism involves the HPDM scaffold inducing macrophage polarization toward the M2 phenotype through the sustained release of polydopamine and Mg^2+^ while activating the TGF-β1 and BMP-2 signaling pathways to promote endothelial cell angiogenesis and pre-osteoblast osteogenic differentiation. These processes further suppress RANKL expression via intercellular communication (Notch signaling pathway), thereby regulating bone metabolic homeostasis.

## CRediT authorship contribution statement

**Caiping Yan:** Writing – original draft, Methodology, Investigation, Data curation. **Fukang Zhu:** Writing – original draft, Methodology, Investigation, Data curation. **Hao Liang:** Investigation, Data curation. **Changxing Liu:** Investigation, Data curation. **Bin He:** Investigation, Data curation. **Taiyou Wang:** Investigation, Formal analysis. **Heling Tan:** Software, Data curation. **Hong Li:** Software, Resources, Data curation. **Dianming Jiang:** Writing – review & editing, Validation, Supervision, Conceptualization. **Bo Qiao:** Writing – review & editing, Validation, Supervision, Conceptualization.

## Availability of data

The datasets used and analyzed during the present study can be obtained from the corresponding author upon reasonable request.

## Funding

This work was supported by the 10.13039/501100001809National Natural Science Foundation of China (82072449); the 10.13039/501100002865Chongqing Municipal Science and Technology Bureau, China (CSTB2024NSCQ-MSX1193).

## Declaration of competing interest

The authors declare that they have no known competing financial interests or personal relationships that could have appeared to influence the work reported in this paper.

## Data Availability

Data will be made available on request.
