# Supplementary material for: 3D-printed nHA/PA66 porous scaffold: Regulating immune balance and vascularization synergistically promotes bone regeneration
Source: Mater Today Bio. 2025 Sep 14;35:102315. doi: 10.1016/j.mtbio.2025.102315 (PMC12478118; doi:10.1016/j.mtbio.2025.102315)
Supplement: Multimedia component 1 [file mmc1.docx]

Supplement information


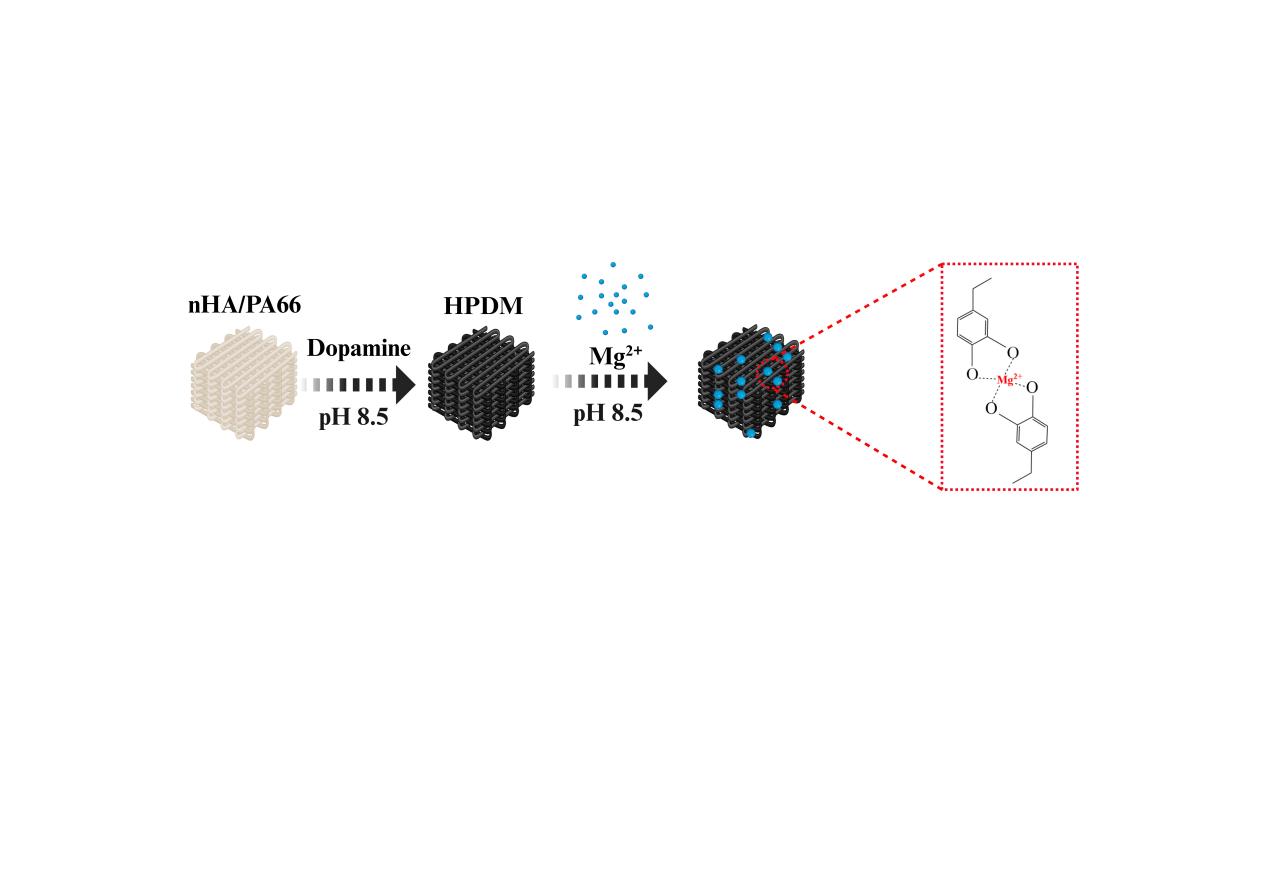


Figure.S1 The porous nHA/PA66 scaffold was modified through a coordination reaction between dopamine and Mg^2+^, resulting in the preparation of the HPDM porous scaffold.

**
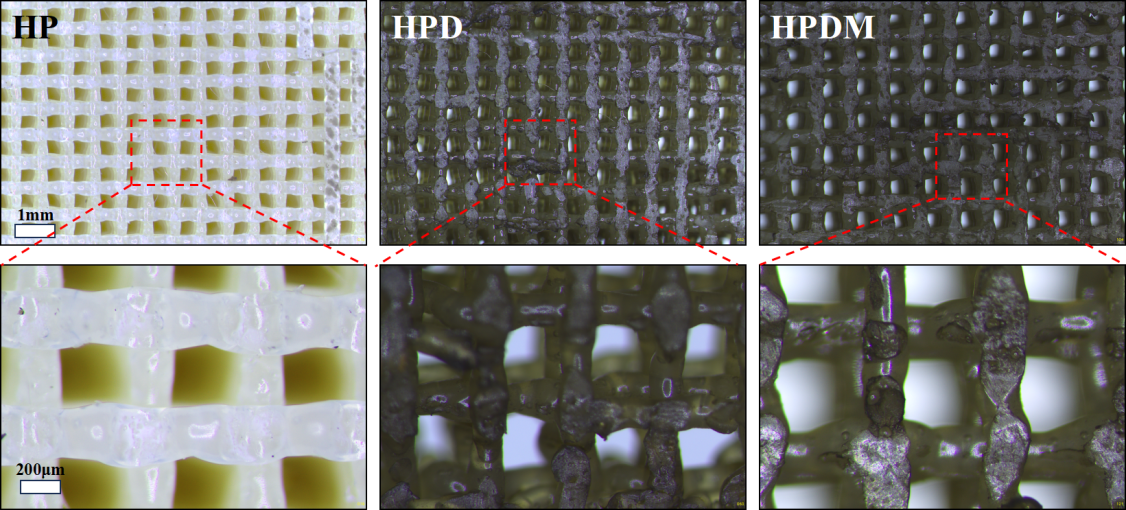
**

Figure.S2 Morphological observation of nHA/PA66 porous scaffolds before and after modification with DOPA and Mg^2+^. From left to right, the groups are HP, HPD, and HPDM.


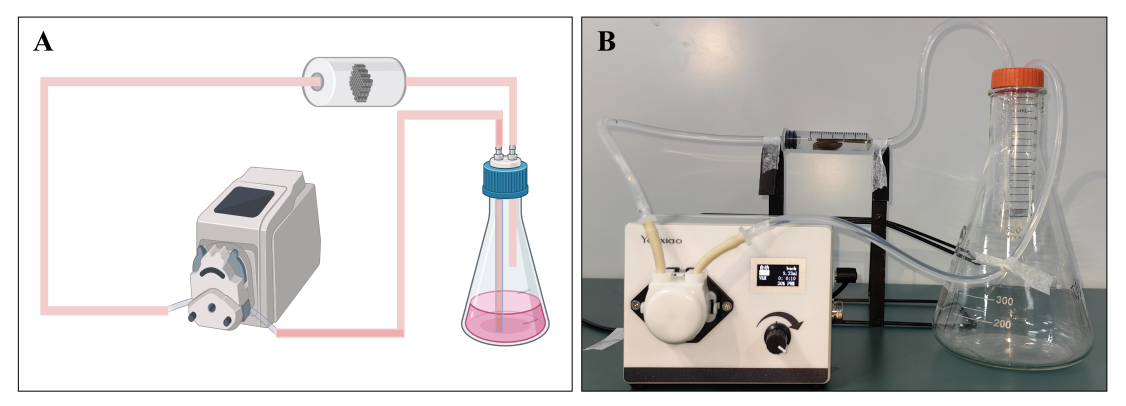


Figure.S3 Schematic (A) and physical drawings (B) of the dynamic degradation experimental device for HPDM scaffolds.


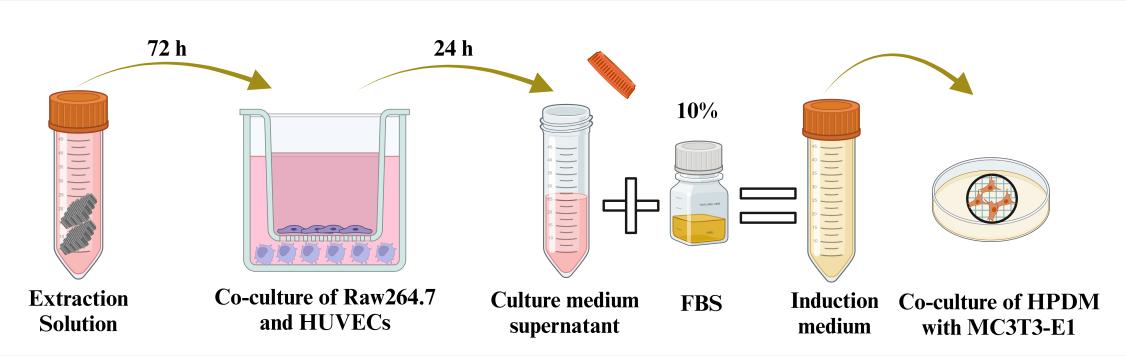


Figure.S4 Schematic diagram of the "induction medium" preparation and co-culture method.


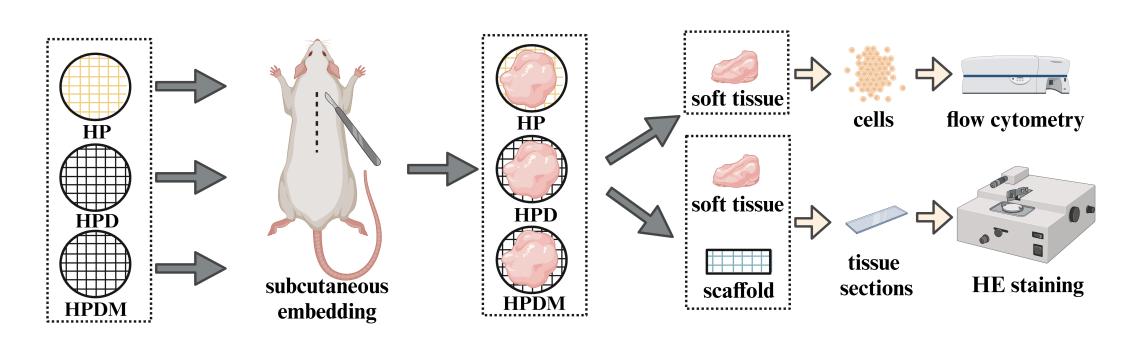


Figure.S5 Schematic diagram of the validated scaffold in vivo study on modulation of macrophage polarization.


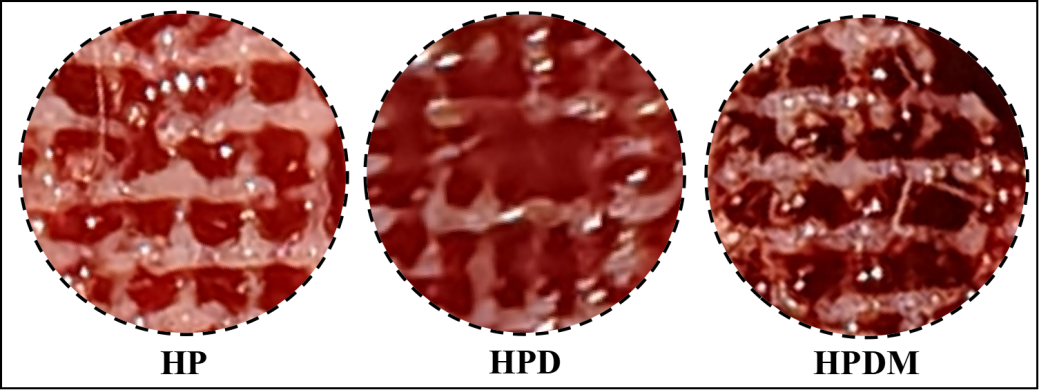


Figure.S6 Modeling and scaffolding of femoral condylar bone defects in rabbits.


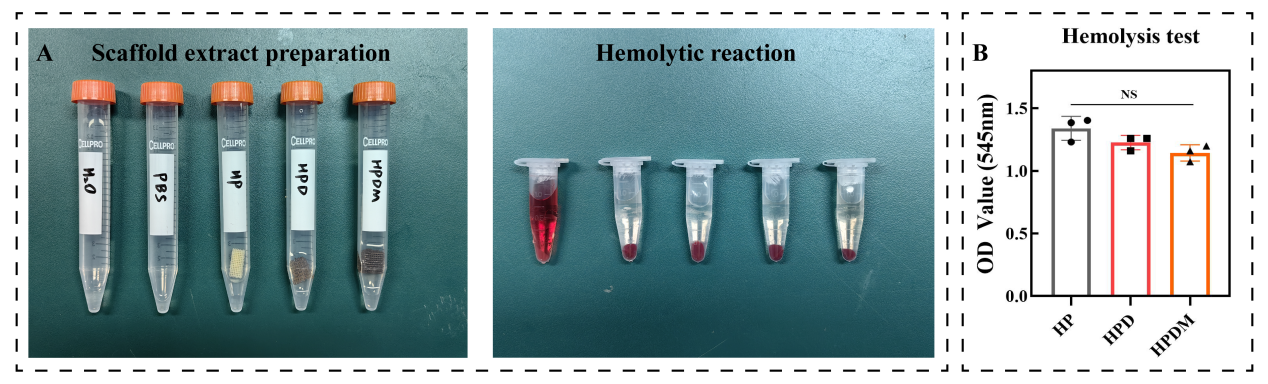


Figure.S7 Results of hemolysis experiments with scaffold extracts (*p < 0.05, NS: no significant difference).


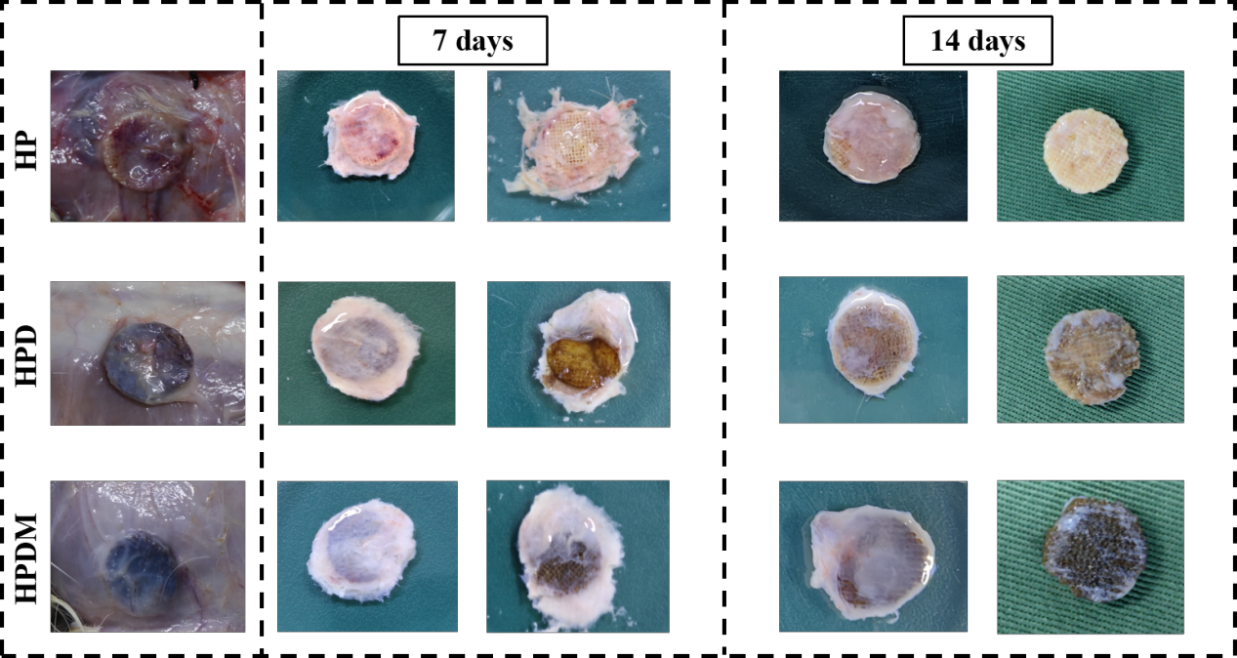


Figure.S8 Proliferation of surrounding tissues at 7 and 14 days post-subcutaneous implantation of various scaffold materials.


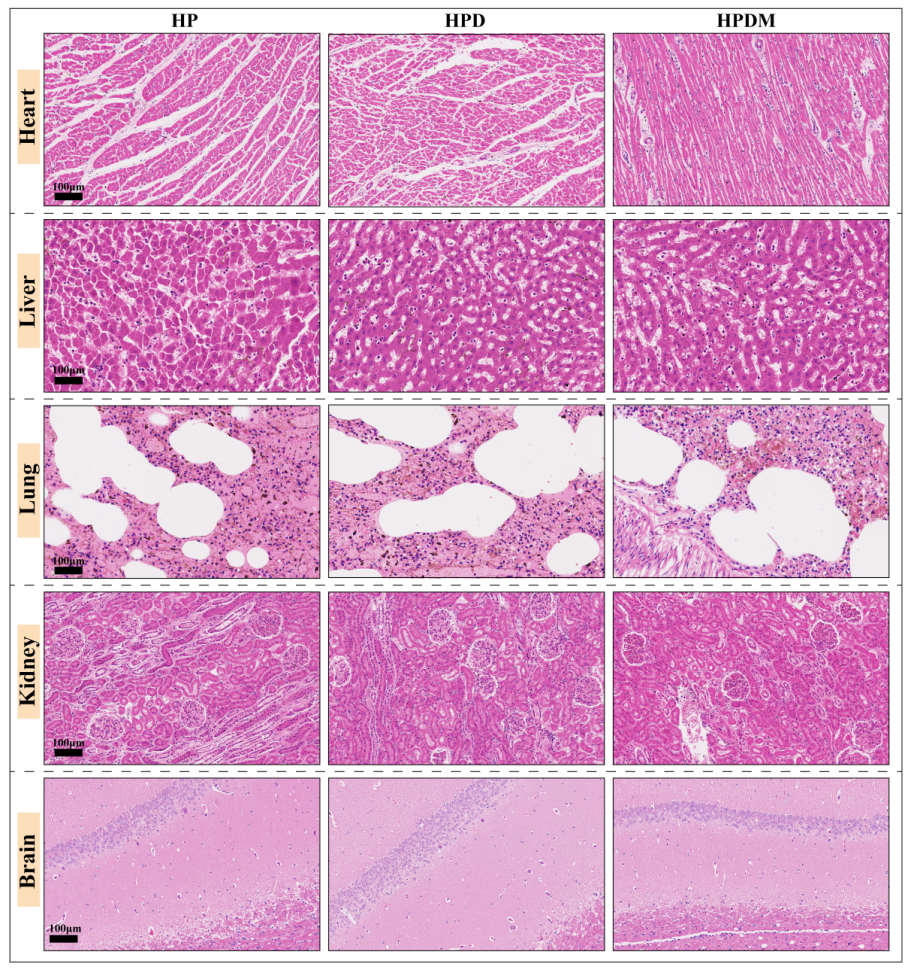


Figure.S9 HE staining results of organ tissues (heart, liver, lungs, kidneys and brain) of New Zealand rabbits at 12 weeks postoperatively.


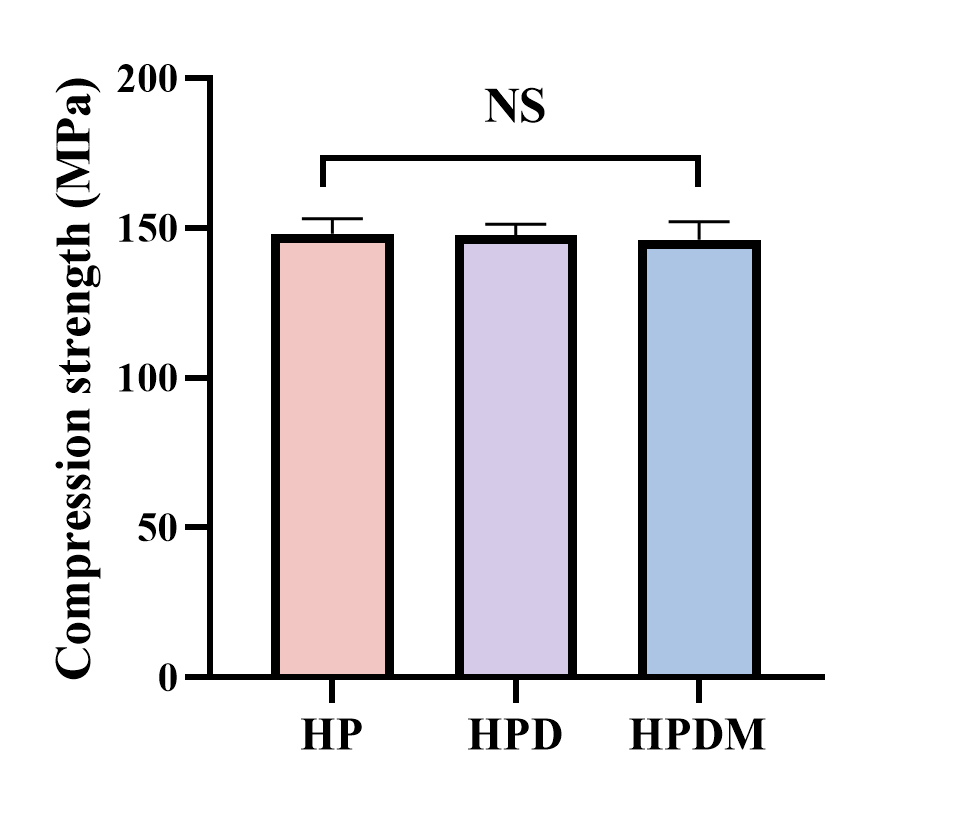


Figure.S10 Compressive strength comparison of HP, HPD, and HPDM porous scaffolds: No significant difference was observed in the compressive strength among the three scaffolds. (One-way ANOVA were used; NS, no significant difference).

**Supplemental dates**：The gene sequencing data involved in this study have been uploaded to the GEO database. The number is: **GSE302075** (https://www.ncbi.nlm.nih.gov/geo/query/acc.cgi?acc=GSE302075)
